# Safety and efficacy of allogeneic umbilical cord blood cells and erythropoietin combination therapy in patients with subacute stroke

**DOI:** 10.1186/s13287-025-04856-8

**Published:** 2025-12-27

**Authors:** Jong Moon Kim, Seyoung Shin, Doyoung Lee, Jee In Choi, Hyeok Gyu Kwon, Sean Soon Sung Hwang, Sun-Mi Cho, Yun-Hee Kim, Jongmin Lee, Hyun Im Moon, Mi Ri Suh, MinYoung Kim

**Affiliations:** 1 Department of Rehabilitation Medicine, CHA Bundang Medical Centre, CHA University School of Medicine, Seongnam, 13496 Republic of Korea; 2 Rehabilitation and Regeneration Research Centre, School of Medicine, Seongnam, 13496 Republic of Korea; 3 Department of Physical Therapy, College of Health Science, Eulji University, Seongnam, 13135 Republic of Korea; 4 Department of Laboratory Medicine, CHA Bundang Medical Centre, CHA University School of Medicine, Seongnam, 13496 Republic of Korea; 5 Department of Physical and Rehabilitation Medicine, Sungkyunkwan University School of Medicine, Suwon, 16419 Republic of Korea; 6 Department of Rehabilitation Medicine, Konkuk University School of Medicine, Seoul, 05030 Republic of Korea; 7 Department of Rehabilitation Medicine, Bundang Jesaeng General Hospital, Seongnam, 13590 Republic of Korea

**Keywords:** Cell therapy, Neuroplasticity, Functional recovery, Neurological, Activities of daily living, Diffusion tensor image

## Abstract

**Background:**

Cell therapy has been proposed as a promising treatment for neurological recovery in patients with stroke. However, a strategy to enhance its efficacy is needed, as its clinical benefits have not yet been demonstrated in clinical trials. This study evaluated the efficacy of combination therapy using allogeneic umbilical cord blood (UCB), a relatively safe therapeutic cell source, and recombinant human erythropoietin (rhEPO) in patients with subacute stroke.

**Methods:**

In this double-blind, randomised controlled trial, we enrolled patients with subacute stroke one to nine months after stroke onset. The patients were divided into three groups: UCB + EPO, UCB, and control. Immune compatibility-matched UCB was intravenously infused once, and rhEPO was administered five times. Safety was evaluated according to the Common Terminology Criteria for Adverse Events (version 5.0), while efficacy was assessed based on changes in activities of daily living, motor and cognitive functions, brain imaging findings, and electroencephalography performed at six months after baseline.

**Results:**

A total of fifteen patients (59.0 ± 10.9 years) were included, with consisting of five patients in each group with comparable demographic data and functional parameters at baseline. Adverse events did not indicate any harmful effects of UCB or rhEPO. After all patients completed the final functional evaluation the UCB + EPO group showed significantly better outcomes than the control group in terms of the total Functional Independent Measure (FIM) (Δ15.00[12.50, 24.50] vs. Δ0.00[-13.00, 3.00], *P* = 0.009), FIM motor subscale (Δ14.00[10.00, 18.50] vs. Δ13.00[0.50, 3.50], *P* = 0.009), and Geriatric Depression Scale (Δ-3.00[-5.00, -2.00] vs. Δ6.00 [-1.00, 18.50], *P* = 0.016) scores. The UCB group showed a marginally non-significant improvement over the control group, without statistical differences in most outcome measures. The brain imaging findings also supported the functional recovery–related effects of UCB therapy.

**Conclusion:**

In conclusion, rhEPO can enhance the efficacy of UCB cells in patients with subacute stroke, without causing harmful effects. This exploratory finding may provide evidence for the potential use of UCB + EPO combination therapy for neurological recovery following stroke.

*Trial registration URL*: https://clinicaltrials.gov/ct2/show/NCT04013646.

**Supplementary Information:**

The online version contains supplementary material available at 10.1186/s13287-025-04856-8.

## Introduction

Cell therapy has emerged as a potential avenue for managing neurological impairment resulting from injuries to the central nervous system, which is known for its limited capacity for self-repair [1]. Among these, stroke is a common and representative disorder that requires advanced approaches for functional recovery [2]. Accordingly, clinical trials using therapeutic cells have steadily increased [3, 4]. Various cell types such as stem cells have been evaluated for their safety and efficacy in patients with stroke [5–7]. Previous reports have highlighted the advantages of allogeneic umbilical cord blood (UCB) as a therapeutic cell source due to its safety and ready availability [8, 9]. UCB contains various stem cells that secrete cytokines, growth factors, and immunomodulatory factors that may be beneficial for neural repair [10, 11]. While the efficacy of UCB has not been extensively validated for stroke, clinical trials have reported its potential therapeutic benefit in other neurological disorders, including cerebral palsy, which shares many pathological mechanisms with stroke [12]. 

While the use of allogeneic UCB cells for regenerative purposes has been shown to be safe [9, 13], the clinical significance of UCB therapy for functional restoration, similar to that of most other stem cell therapies, has not been clearly recognised [1, 14]. In previous clinical trials that applied UCB for cerebral palsy, our research team observed the potentiation of therapeutic effects by combining UCB with recombinant human erythropoietin (rhEPO) administration [15]. Notably, rhEPO has been shown to exerts neuroprotective effects against ischaemic stroke by enhancing angiogenesis and neurogenesis [16, 17]. Moreover, rhEPO attenuates microglial activation and promotes beneficial polarisation, which may facilitate recovery from stroke-related neuronal damage, in which neuroinflammation persists [18]. A previous in vivo study revealed a significant synergistic effect of UCB and rhEPO combination therapy in a subacute stroke model by demonstrating augmented recovery of neurobehavior with compatible neuroprotective and angiogenic effects in brain tissue [19]. 

A phase I trial by Laskowitz et al. reported positive results for UCB infusion in patients with acute ischaemic stroke in (modified Rankin score, National Institutes of Health Stroke Scale, and Barthel Index), though similar findings were not observed, in phase II, potentially due to COVID-19-related disruptions during the study period [20, 21]. This study verified the safety of allogeneic UCB in patients with stroke and indicated its potential therapeutic application. However, UCB infusion was administered within 5–9 days of stroke onset, likely during the inflammatory response phase. Based on previous findings in children with cerebral palsy, UCB may exert its therapeutic effects after acute inflammation has subsided [22]. 

Therefore, the current clinical study aimed to investigate the therapeutic efficacy and safety of allogeneic UCB + rhEPO combination therapy in post-stroke patients during the subacute phase, when patients were medically stable but the injured tissue was affected by post-stroke changes [23]. The protocol was based on previous trials that considered immune compatibility and cell number criteria when selecting UCB units, and the administration of immunosuppressants and rhEPO [23, 24]. Follow-up to evaluate therapeutic efficacy in each patient lasted six months. All adverse events (AEs) were monitored for one year. The primary aim of this pilot trial was to evaluate the safety and feasibility of allogeneic UCB + rhEPO administration. Functional and cognitive outcomes were included as exploratory, and hypothesis-generating secondary endpoints. Additionally, diffusion tensor tractography, electroencephalography (EEG), and inflammatory cytokine activity were performed to investigate changes in patients, particularly in the brain tissue, that may be associated with functional outcomes.

## Materials and methods

### Participants

The inclusion criteria were patients with stroke aged 20 years or older who had definite hemiplegia involving upper extremity paralysis from 30 days to nine months after stroke onset, with unilateral supratentorial lesion of infarct or haemorrhage, and who provided written informed consent for study participation directly or via their legal representatives. The exclusion criteria were medical instability or abnormal blood laboratory test results, including chemistry and blood cell counts, immunodeficiency or malignant tumours not in complete remission for over 10 years, and side effects to rhEPO or tacrolimus (details in Supplemental Methods).

The study protocol and informed consent forms were approved by the Institutional Review Board of the CHA Bundang Medical Centre (No. 2018-12-030-073) and the Ministry of Food and Drug Safety of Korea (No. 32153). Informed consent was obtained from all participants or their legal representatives before their voluntary participation. Allogeneic UCB units were supplied by the CHA Cord Blood Bank after obtaining approval from the Korean Network for Organ Sharing at the Korea Centres for Disease Control and Prevention, Ministry of Health and Welfare. This clinical trial was registered at ClinicalTrials.gov (NCT 04013646).

### Study design and masking

This clinical trial was designed and conducted as a placebo-controlled, double-blind study at a university hospital. Among 16 patients that were screened, 15 underwent randomisation and completed the study, with 5 patients in each of the following groups: the UCB + EPO group that received UCB and rhEPO; the UCB group that received UCB and placebo rhEPO; and the control group that received placebo UCB and placebo rhEPO. To account for potential dropout, the target number of enrolled participants was set to 16.

As a pilot study, no a priori power calculations or formal sample size estimations were undertaken. The primary aim of the trial was to evaluate safety and feasibility, with efficacy outcomes analysed in an exploratory manner. Randomization was performed by an independent researcher not involved in patient recruitment, treatment, or assessment, using the SPSS random number generator in accordance with the CONSORT 2010 guidelines [25]. Allocation concealment was ensured by providing only coded labels to the pharmacist, who prepared the study medication or placebo in indistinguishable packaging. Nurses administered the interventions according to the code, while patients, treating physicians, and outcome assessors remained blinded throughout the study (Supplementary Fig. 1).

No interim analyses were planned or conducted. Although stopping criteria were predefined, no conditions requiring study termination occurred.

### Intervention protocol

Allogeneic UCB units were obtained from donors who had provided informed consent Through the CHA Cord Blood Bank. Selection criteria for UCB units were at least four out of the six human leukocyte antigens ((HLA)-A, -B, and -DRB1) matches at high resolution and ABO blood type compatibility following the general transfusion principle. For each patient, the administered number of total nucleated cells (TNC) was required to be at least 2 × 10^7^/kg of body weight. As a single UCB unit generally contained an insufficient number of cells for adult recipient, administration of multiple units was permitted to meet the required dose. Patient-level details including total TNC, TNC per kg, viability, blood type, and HLA mismatches are summarized in Supplementary Table 3. Each unit was washed to remove dimethyl sulfoxide according to the institutional protocol and assessed for cell survival [26]. UCB cells were then intravenously infused individually by the principal investigator Blood pressure, pulse rate, body temperature, and peripheral oxygen saturation (SpO2) were monitored continuously from 30 min before to 30 min after infusion. For patients receiving UCB, tacrolimus (Tacrobell^®^, ChongKunDang Pharmaceutical Corp., Republic of Korea) was orally administered as an immunosuppressant for one week starting one day prior to UCB administration. This regimen aimed to prolong the survival of UCB cells by reducing immune-mediated clearance and to prevent immune reactions to the administered proteins, in accordance with previous paediatric protocol [27]. The dosage of the regimen was 0.06 mg/kg twice daily, and the target blood concentration was adjusted to 5–20 ng/mL [28]. 

rhEPO (Esposis^®^, Daewoong Pharmaceutical Co., Seoul, Republic of Korea) was intravenously injected twice per week at 500 IU/kg, for a total of five weeks. If haemoglobin levels exceeded 13.6 g/dL before EPO administration, phlebotomy was performed up to two times according to the protocol until confirmation of the level below 13.6 g/dL.

All participants received standard rehabilitation treatment and were closely monitored for any AEs during their hospitalisation period of at least one month. Autologous peripheral blood diluted to 10% with normal saline was used as the placebo. The placebo rhEPO was the vehicle for Eposis^®^ and the placebo tacrolimus was the vehicle for Tacrobell; each was manufactured and packaged by the manufacturing company. All placebo materials were indistinguishable and identifiable only by serial numbers.

### Safety assessment

To evaluate safety-which is the primary outcome of this study-all AEs, as defined in the Common Terminology Criteria for Adverse Events version 5.0, were monitored until 12 months after UCB and EPO administration [29]. Serious AEs were defined as (i) death, (ii) requirement for inpatient hospitalisation or prolongation of hospitalisation, (iii) life-threatening AEs, or (iv) persistent or significant disability. Newly developed or worsening symptoms and abnormal vital sign, physical examination, electrocardiogram, and blood laboratory tests findings were considered AEs.

### Functional assessment

**Fig. 1:**
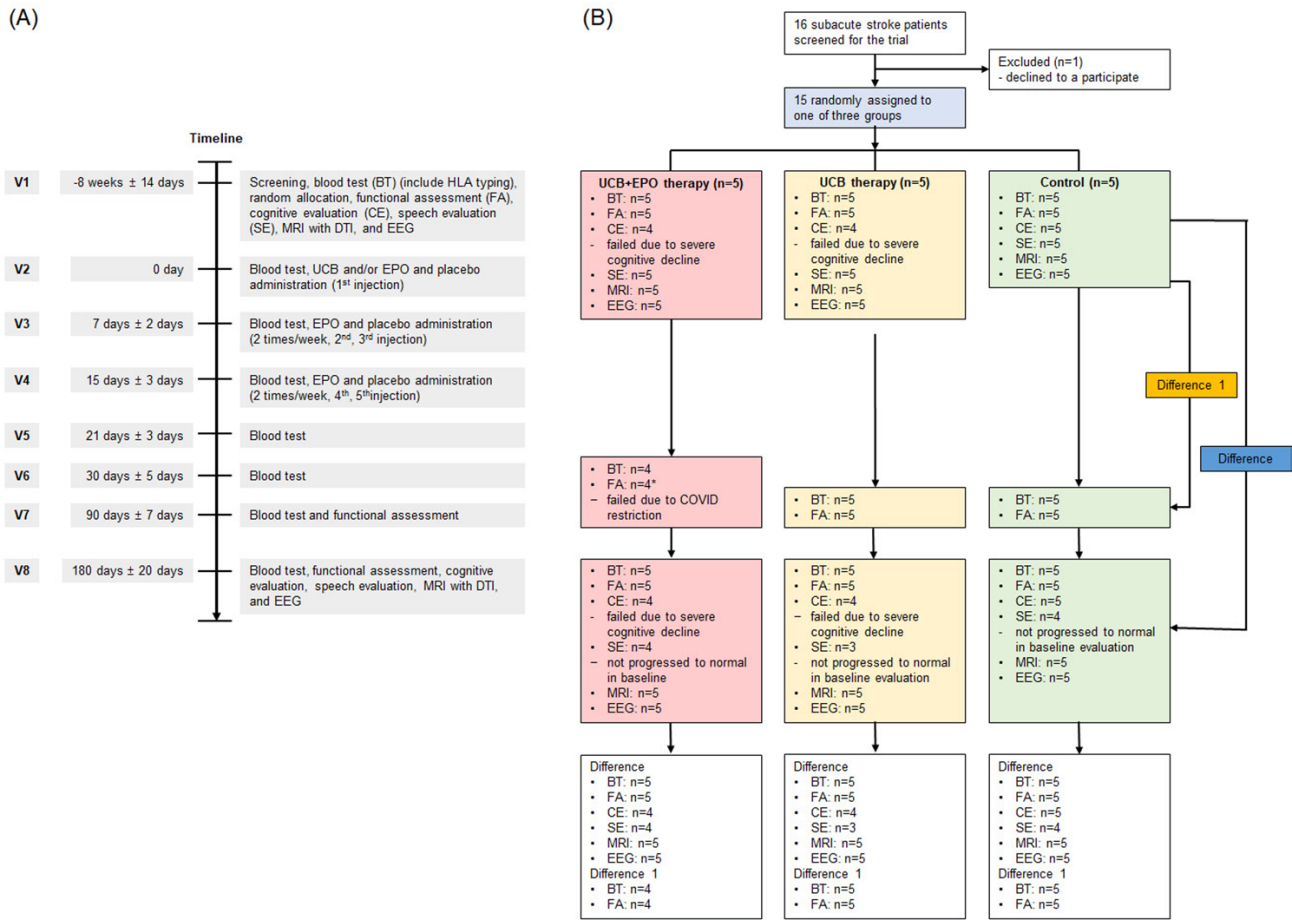
The timeline of the study. Outcome variables were BT, FA, CE and SE performed 180 days after baseline assessment (Difference). At 90 days after the baseline evaluation, the results of BT and FA were confirmed (Difference 1). Some patients were unable to perform CE due to cognitive decline, and patients whose SE was measured as normal at baseline assessment did not receive follow-up SE. One patient in the UCB+EPO group was unable to undergo a 90-day evaluation due to COVID-19 infection. *HLA* Human Leukocyte Antigen, *MRI* Magnetic Resonance Imaging, *DTI* Diffusion Tensor Imaging, *EEG* Electroencephalogram, *UCB* Umbilical Cord Bloo, *EPO* recombinant human Erythropoietin, *BT* Blood Test, *FA* Functional Assessment, *CE* Cognitive evaluation, *SE* Speech Evaluation

Exploratory outcomes were assessed by changes in the total score of the Functional Independence Measure (FIM), which represents the ability to perform activities of daily living (ADL), as well as changes in the motor and cognitive FIM subscale scores at three and six months after therapy [30]. Scores from the following evaluation tools were used as secondary outcome variables: the National Institutes of Health Stroke Scale was employed determine neurological impairment status [31]; the sum of numerically converted scores from the joints according to the Medical Research Council scale (MRC) was used to evaluate muscle strength in the paralytic limbs (Supplementary Table 1) [32–34]; the total scores of the upper extremity Fugl-Meyer Assessment (FMA) [35] and Manual Functional Test [36] were used as upper extremity function measurements; gross motor ability was assessed using the Berg Balance Scale (BBS) [37] and Trunk Imbalance Scale [38]. The reliability of these functional outcome measurements among raters was established among all clinical evaluators prior to study initiation with an inter-rater intra-class correlation coefficient of > 0.9, which was a re-established annually [39]. These efficacy outcome measurements were performed at baseline and at three and six months after therapy (Fig. 1A).

Cognitive evaluations included the Mini-Mental Status Examination (MMSE) [40], Montreal Cognitive Assessment (MoCA) [41], Clinical Dementia Rating (CDR) [42], Global Deterioration Scale [43], Rey-Kim Memory Quotient [44], and Wechsler Adult Intelligence Scale-IV (WAIS-IV) [45] were used. Language ability was assessed using the Western Aphasia Battery [46]. The evaluation tools used in this trial have been validated for the Korean population. Cognitive and speech abilities were evaluated by a corresponding expert, a clinical psychologist, and a speech-language pathologist. With the exception of the MMSE and MoCA which were evaluated alongside other functional assessments, these were followed up six months after later. Patients with normal cognition or language ability in the WAIS-IV and WAB before the trial did not undergo a repeated baseline study.

### Laboratory evaluation

In addition to routine blood chemistry and complete blood cell counts at baseline (D-1) and at 1, 7, 15, and 30 days after therapy, serum tacrolimus levels were also measured one day after UCB infusion. To maintain the double-blind study design, random values were provided by the Laboratory Medicine Department (Supplementary Fig. 1). For biomarker assays related to therapeutic mechanisms, peripheral blood mononuclear cells and plasma were isolated from whole blood samples of each patient. Quantitative real-time polymerase chain reaction (qRT-PCR) for inflammation associated cytokines, namely TNF-α, TGF-β, IL-1β, and IL-8 were assayed. In addition, 80 proteins were tested to evaluate changes in cytokine levels using the Human Cytokine Antibody Array C5 (RayBiotech, Inc.) [47] for selected cases (details in Supplemental Methods).

### Brain MRI and EEG

At baseline and six months post-treatment, patients underwent, routine brain magnetic resonance imaging (MRI) (GE Healthcare, Milwaukee, WI, USA) with diffusion tensor imaging (DTI) for further analysis. After preprocessing the DTI data, tractography was performed and fractional anisotropy (FA) values were measured for the corticospinal tract (CST), somatosensory tract (SST), cingulum, and arcuate fasciculus (AF) [48, 49]. All MRI scans were read and reported by an expert neuroradiologist, and DTI analyses were performed by a diffusion tractography specialist (Kwon HG), was blinded to the group assignments (details in Supplemental Methods).

In addition, the patients underwent EEG at baseline and at six months follow-up. Resting-state EEG data were recorded for 3 min with both eyes open and closed. EEG segments with eyes closed were used for group analysis. EEG preprocessing and intra- and inter-group analyses were conducted using the EEG analysis platform iSyncBrain (iMediSync Inc., Republic of Korea, https://isyncbrain.com) approved by the US FDA [50]. We compared changes in EEG power from baseline to six months after the intervention across the three groups. For each patient, the difference in power between baseline and six months was calculated for the alpha1, alpha2, and theta bands. Group-level statistical comparisons were then performed to examine intergroup differences in these change values. Mean differences were calculated for at baseline and six months after therapy.

### Statistical analysis

Statistical analyses were performed using SPSS software version 21.0 (SPSS, Inc., Chicago, IL, USA). All randomised participants were included in the primary analysis according to the intention-to-treat principle and analysed in the groups to which they were originally assigned, regardless of protocol adherence. Participants with missing data were excluded from the analysis, and only those with complete data were included.

The normality of data distributions was assessed using the Shapiro–Wilk test. Because most variables were not normally distributed and the sample size was small, nonparametric tests were applied. Baseline demographic and clinical characteristics were compared using the Kruskal–Wallis test for continuous variables and the Fisher’s exact test for categorical variables. For safety assessment, AEs across groups were compared using the Fisher’s exact test. Changes in functional outcome measures from baseline to six-month post-therapy were compared among the groups using the Kruskal-Wallis test for three group comparison and Mann-Whitney U test with Bonferroni correction for post-hoc analysis. Differences in time-group interactions over time were examined using repeated-measures ANOVA (RM-ANOVA), which were presented as exploratory analyses only, given the small sample size and limited statistical power. Statistical significance was set at *P* < 0.05 statistically significant. Results with *P*-values between 0.05 and 0.1 were considered marginally non-significant.

## Results

**Fig. 2:**
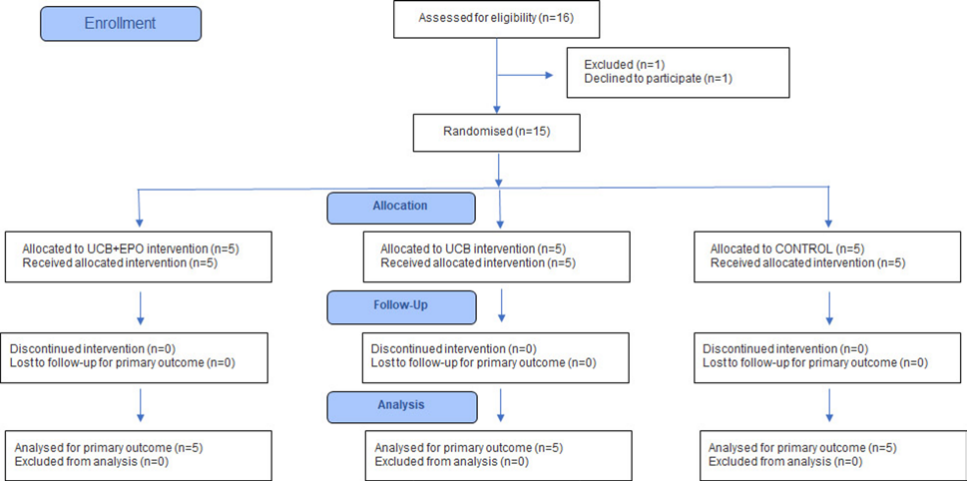
CONSORT Flow Diagram. Flow diagram of the progress through the phases of a randomised trial of three groups (that is, enrolment, intervention allocation, follow-up, and data analysis). *UCB* Umbilical Cord Blood, *EPO* recombinant human Erythropoietin

From December 2019 to June 2021, 16 patients were screened of whom 1 was excluded who declined to participate prior to randomisation. The remaining 15 patients were enrolled and randomly assigned to the UCB + EPO (*n* = 5), UCB (*n* = 5), or control (*n* = 5) groups (Fig. 2, Supplementary Table 2). Supplementary Table 3 shows the composition of the unrelated allogeneic UCB units administered to each participant. All randomised participants (*n* = 15) completed the trial until the final follow-up evaluation and were included in the analysis, with both the intervention and comparator delivered as per the study protocol. All participants continued to receive standard care throughout the study period, including the usual medications and rehabilitation services, as clinically indicated. The mean post-stroke duration was 78 days [IQR 64–116 days]. There were no differences between the groups in terms of demographic characteristics or functional status, which showed typical features of motor involvement in supratentorial stroke (Table 1). Neurological impairment status was deemed moderate, with a median NIHSS scores of 6.00 [3.00, 13.00]. Regarding cognition, the median MMSE scores was 27.00 [15.00, 30.00], indicating mild or no cognitive impairment [51]. The median FMA score of the upper extremity on the affected side was 9.00 [4.00, 15.00], indicating moderate to severe contralateral upper limb paralysis [52]. Baseline functional assessments were performed within five weeks prior to therapy, without differences between the groups. None of the participants in the UCB + EPO group met the criteria for phlebotomy. However, in the control group, two participants met the haemoglobin threshold requiring phlebotomy. As the control group did not receive UCB or rhEPO, a sham phlebotomy was performed. Sham phlebotomy was performed with dry needle puncture under sight-shield conditions using a curtain. Accordingly, participants received the desired doses of rhEPO. No important changes to the trial design, methods, or outcomes were made after the trial commenced.

**Table 1:** Baseline demographic and functional characteristics of the study enrolled patients

Variables	Total (*n* = 15)	Group			*P* value
		UCB + EPO (*n* = 5)	UCB (*n* = 5)	Control (*n* = 5)	
Sex (n)					
Male	11	3	3	5	0.256
Female	4	2	2	0	
Age, year	57.00[49.00,72.00]	57.00[53.00,72.00]	58.00[54.00,58.00]	49.00[49.00,75.00]	0.982
Post-stroke duration, days	78.00[64.00,116.00]	71.00[64.00,80.00]	78.00[64.00,90.00]	122.00[71.00,138.00]	0.250
Type (n)					
Ischemic	7	3	3	1	0.343
Hemorrhagic	8	2	2	4	
Hemiplegic side (n)					
Right	6	3	2	1	0.435
Left	9	2	3	4	
FIM Total score	82.00[51.00,88.00]	86.00[43.00,87.00]	82.00[73.00,83.00]	79.00[60.00,89.00]	0.878
FIM Motor subscale	50.00[29.00,55.00]	51.00[26.00,60.50]	50.00[36.00,52.50]	44.00[36.50,67.50]	0.960
MMSE	27.00[15.00,30.00]	22.00[19.00,29.00]	27.00[21.00,30.00]	30.00[15.00,30.00]	0.784
NIHSS	6.00[3.00,13.00]	9.00[4.00,29.00]	6.00[6.00,9.00]	8.00[6.00,9.00]	0.953
FMA on affected side	9.00[4.00,15.00]	12.00[4.00,22.00]	8.00[5.50,11.50]	10.00[5.50,30.50]	0.849
BBS	33.00[20.00,41.00]	31.00[4.00,37.00]	24.00[23.00,35.00]	36.00[33.00,42.00]	0.645

Control group refers to the group that did not receive UCB or EPO therapy

UCB group refers to the group that received only UCB therapy. The UCB + EPO group refers to the group that received both UCB and EPO therapy

*, The duration from the occurrence of a stroke to the UCB injection day

Continuous variables were demonstrated as median [Q1, Q3] and categorical variables were demonstrated as number

*P*-values from Kruskal–Wallis test or Fisher’s exact test

*UCB* Umbilical Cord Blood, *EPO* Erythropoietin, *FIM* Functional Independence Measure, *FAC* Functional Ambulation Category, *K-MMSE* Korean version of Mini-Mental State Exam, *NIHSS* National Institutes of Health Stroke Scale, *FMA* Fugl-Meyer Assessment, *BBS* Berg Balance Scale

### Safety assessment

One serious AE, hydrocephalus, occurred in the UCB group eight months after stroke onset, whereas intracerebral haemorrhage in the right basal ganglia and intraventricular haemorrhage occurred five months after UCB administration. Considering the possibility of hydrocephalus development as a complication of cerebral haemorrhage and the post-intervention duration, this event was determined to be unrelated to the therapy. Six AEs were reported in two patients in the control group, six events occurred in four patients in the UCB group, and nine events were detected in four patients in the UCB + EPO group. No significant differences in the number of AEs were observed between groups. All AEs were determined to be unrelated to the therapy, except for one case of ‘chest discomfort’ in the UCB + EPO group, which occurred on the day after UCB infusion, which was classified as being ‘possibly related’ to the therapy. Monitored vital signs, including oxygen saturation, were within normal limits during the entire process. Electrocardiographic findings did not suggest any cardiac events. All AEs resolved after the appropriate treatment (Table 2).

**Table 2:** Adverse effect according to common terminology criteria for adverse events (CTCAE) after therapy

Variables	Group			Total (*n* = 15)	*P* value
	UCB + EPO (*n* = 5)	UCB (*n* = 5)	Control (*n* = 5)		
Serious adverse event ^a^					
Hydrocephalus ^b^		1 (V7) ^d^		1	0.343
Other adverse events					
Chest pain - cardiac	1 (V2) ^f^			1	0.343
Headache	1 (V2) ^f^	1 (V4) ^e^		2	0.562
Pain in extremity (CRPS)	2 (V3, V3) ^e^			2	0.099
Pain in extremity (Shoulder)	2 (V3, V3) ^e^			2	0.099
Fracture (5th toenail defect)		1 (V3) ^d^		1	0.343
Sore throat			1 (V3) ^d^	1	0.343
Allergic rhinitis			1 (V3) ^d^	1	0.343
Urinary tract infection	1 (V5) ^e^	1 (V7) ^e^		2	0.562
Gastritis			1 (V1) ^e^	1	0.343
ALT/AST increased		1 (V5, V8) ^c^		2	0.343
Hypertriglyceride			1 (V3) ^e^	1	0.343
Hyperglycemia			2 (V3, V7) ^e^	2	0.099
Depression	1 (V5) ^e^	1 (V8) ^e^		2	0.562
Edema limbs	1 (V5) ^e^			1	0.343
Total	9	6	6	21	

Unknown event was confirmed (chest discomfort) in 1 person of UCB + EPO group. a The day after UCB injection, the patient had chest discomfort and headache, vital signs were tolerable, and the EKG showed the same findings as before. After taking painkiller (tridol), symptoms improved 30 min after onset

Serious adverse events were defined as any event, resulting in death, life-threatening, requiring hospitalization or prolongation of hospital stay. A serious adverse event was confirmed in one person in the UCB group (b hydrocephalus), but it was an unrelated event. Hydrocephalus corresponded to CTCAE grade 3, and the rest corresponded to grade 1 or 2. AST/ALT elevation occurred 2 times in one subject (c ALT/AST elevation). Relationships with the intervention were shown as d unlikely, e non-related or f possible related. The source of terminology was Medical Dictionary for Regulatory Activities (MedDRA) 21.1

The period of occurrence was divided as follows. V1: Before the therapy (D-1, Tacrolimus administration starts date), V2: On the day of therapy (D-day) ~ D + 6 days, V3: D + 7 ~ 14, V4: D + 15 ~ 20, V5: D + 21 ~ 29, V6: D + 30 ~ 89, V7: D + 90 ~ 179, V8: After D + 180

*P*-values from Fisher’s exact test

*UCB* Umbilical Cord Blood, *ALT* Alanine aminotransferase, *AST* Aspartate aminotransferase, *CRPS* Complex Regional Pain Syndrome

### Functional assessment

**Fig. 3:**
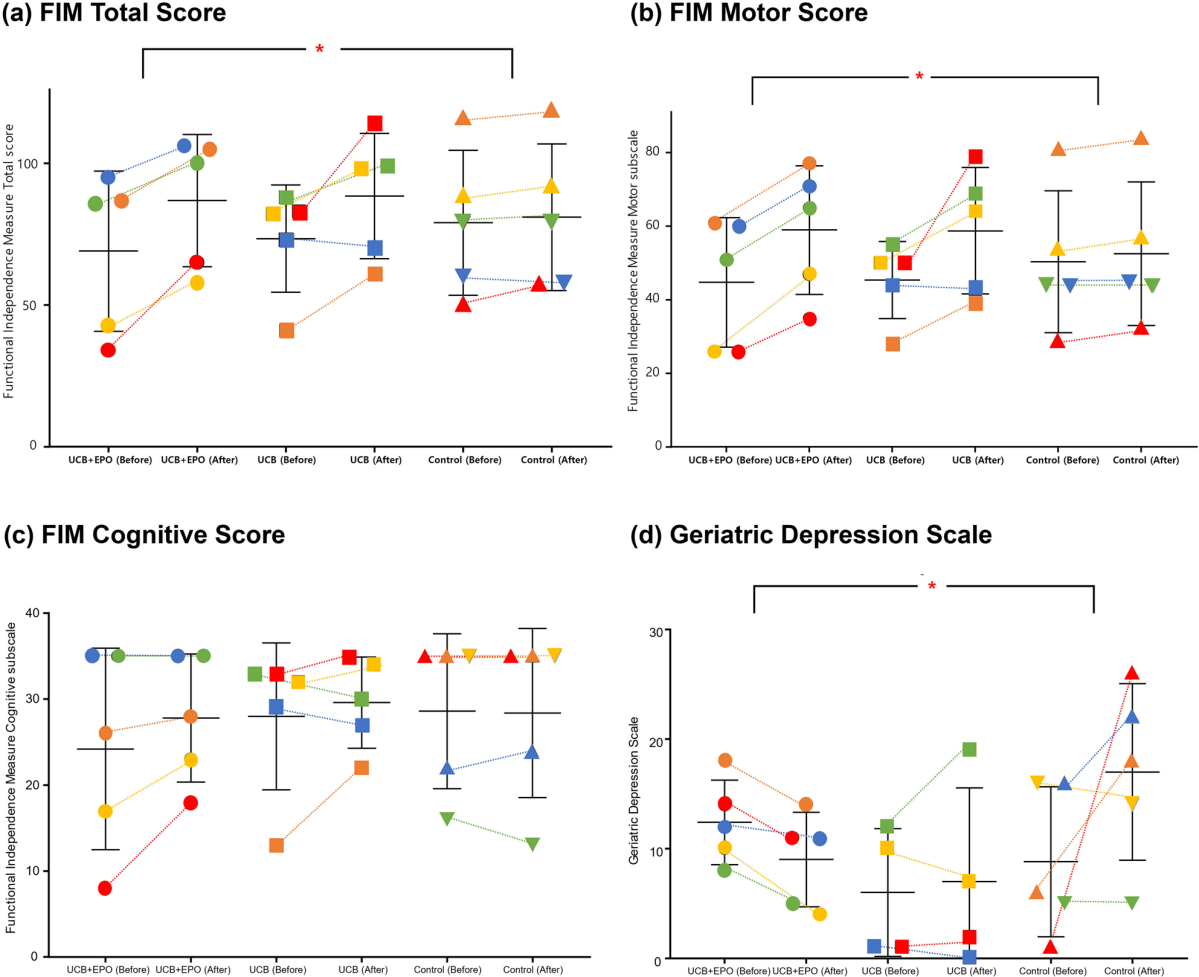
Comparison of functional changes from baseline to 180 days after therapy. In each group (*n*=5), the baseline measurement value and the change value after 180 days are indicated. Asterisks indicate significant difference in outcome scores between two groups based on group comparison analysis (Bonferroni-adjusted *P*<0.016) (Mann-Whitney U test) following Kruskal-Wallis test. * *P*<0.0167. (**a**–**c**) Motor/cognitive subscale and total score of functional independence measure, (**d**) Geriatric Depression Scale. The colour and shape of each subject were determined based on their baseline FIM total score (from red for the highest increment, orange, yellow, green, and blue the lowest increment in order), and the same symbols were used consistently throughout the subsequent figures. *UCB* Umbilical Cord Blood, *EPO* Erythropoietin

Significant differences in FIM score changes were observed between the groups over time. Three months after the intervention, no significant differences were observed between the groups. In contrast, at six months after the intervention, the UCB + EPO group showed significantly greater improvements in the FIM total scores than those of the control group (Δ15.00 [12.50, 24.50] vs. Δ0.00 [-13.00, 3.00], *P* = 0.009). In particular, significant improvements in the motor subscore of FIM were noted (Δ14.00 [10.00, 18.50] vs. Δ3.00 [0.50, 3.50], *P* = 0.009), indicating a potential treatment-related effect. Furthermore, the depression scale (GDS-d) also showed a nominally significant difference between the UCB + EPO and control groups (Δ-3.00 [-5.00, -2.00] vs. Δ6.00 [-1.00, 18.50], *P* = 0.016) (Table 3). The increase in BBS scores at 6 month was greater in the UCB + EPO group compared to the control group (Δ12.00 [6.50, 16.50] vs. Δ2.00 [-0.50, 3.50], *P* = 0.036); however, this difference did not remain significant after Bonferroni-adjusted pairwise comparison. At 3 months, the MoCA score showed a significant omnibus difference across groups (Kruskal–Wallis *P* < 0.05). However, none of the pairwise comparisons remained significant after Bonferroni correction. For the CDR at 6 months, an overall group difference was detected (Kruskal–Wallis *P* < 0.05), but post-hoc pairwise tests did not reveal significant contrasts after correction (Table 3; Figure. 3). Repeated-measures ANOVA indicated no significant time-group interactions across time points (Supplementary Table 4). One participant in the control group had a post-stroke duration of > 200 days; however, when the same analyses were conducted excluding this individual, the statistical significance of the changes in functional outcomes remained unchanged.

**Table 3:** The comparison of changes in functional outcomes between groups

Variables	Total (*n* = 15)	Group			^a^*P*-value			^b^*P*-value
		UCB + EPO (*n* = 5)	UCB (*n* = 5)	Control (*n* = 5)	UCB + EPO vs. Control	UCB + EPO vs. UCB	UCB vs. Control	
FIM total								
Δ3months-base	6.00[1.00,11.00]	10.00[-17.00,13.50]	6.00[-1.00,22.50]	2.00[-1.00,4.50]	0.116	0.917	0.249	0.275
Δ6months-base	11.00[0.00,18.00]	15.00[12.50,24.50]	16.00[4.00,25.50]	0.00[-13.00,3.00]	0.009*	0.916	0.075	< 0.05*
FIM motor								
Δ3months-base	7.00[1.00,11.00]	10.00[-9.50,12.50]	10.00[1.50.20.50]	2.00[-1.00,4.50]	0.141	0.834	0.116	0.202
Δ6months-base	11.00[0.00,18.00]	14.00[10.00,18.50]	14.00[5.00,21.00]	3.00[0.50,3.50]	0.009*	0.832	0.115	< 0.05*
FIM cog								
Δ3months-base	0.00[0.00,2.00]	0.00[-8.50,2.00]	2.00[-5.50,2.00]	0.00[0.00,0.00]	0.519	0.737	0.572	0.784
Δ6months-base	0.00[0.00,2.00]	0.00[2.00,8.00]	2.00[-2.50,5.50]	0.00[-1.50,1.00]	0.118	0.458	0.519	0.328
NIHSS								
Δ3months-base	-1.00[-1.00,0.00]	0.00[-11.00,0.00]	0.00[-2.50,0.00]	-1.00[-1.00,-0.50]	0.824	0.638	0.488	0.784
Δ6months-base	-1.00[-3.00,0.00]	-1.00 [-7.50,0.00]	0.00 [-2.50,0.00]	-1.00 [-2.00,-0.50]	0.827	0.371	0.435	0.616
MMT affected side								
Δ3months-base	125.00 [0.00,300.00]	100.00 [-62.50,367.50]	130.00 [47.50,142.50]	125.00 [-5.00,330.00]	0.754	0.917	0.917	0.970
Δ6months-base	140.00 [15.00,365.00]	295.00 [77.50,392.50]	70.00 [-5.00,257.50]	145.00 [-20.00,257.50]	0.249	0.347	0.753	0.467
BBS								
Δ3months-base	3.00[0.00,10.00]	8.00[1.50,11.50]	8.00[0.50,14.50]	2.00[-1.00,2.50]	0.073	0.753	0.207	0.202
Δ6months-base	4.00[1.00,12.00]	12.00 [6.50,16.50]	9.00 [0.50,15.00]	2.00 [-0.50,3.50]	0.036	0.402	0.209	0.101
TIS								
Δ3months-base	2.00[0.00,3.00]	2.00[-1.50,5.00]	1.00[0.00,3.00]	2.00[0.00,3.00]	0.914	0.916	0.831	0.982
Δ6months-base	2.00 [0.00,5.00]	2.00 [1.00,11.00]	0.00 [-0.50,3.50]	2.00 [-1.00,4.00]	0.280	0.164	0.748	0.332
MFT affected side								
Δ3months-base	0.00[0.00,5.00]	5.00[1.25,5.75]	0.00[0.00,1.50]	0.00[0.00,3.50]	0.281	0.095	0.700	0.258
Δ6months-base	2.00 [0.00,5.00]	5.00[1.00,10.00]	1.00 [0.50,1.50]	3.00 [0.00,5.50]	0.340	0.110	0.452	0.254
FMA affected side								
Δ3months-base	3.00[0.00,13.00]	10.00[2.25,17.00]	1.00[0.00,13.00]	2.00[0.00,4.50]	0.085	0.172	0.746	0.189
Δ6months-base	6.00 [3.00,16.00]	12.00[4.00,22.00]	12.00[2.50,16.50]	5.00 [1.50,9.50]	0.248	0.673	0.344	0.439
MMSE								
Δ3months-base	0.00[-0.25,1.25]	1.50[0.25,8.00]	0.00[-1.50,2.00]	0.00[-1.50,0.00]	0.030	0.159	0.480	0.082
Δ6months-base	1.00 [-1.00,3.00]	2.00[0.50,8.00]	2.00[-1.00,4.00]	-1.00[-3.00,0.50]	0.027	0.599	0.141	0.081
MoCA								
Δ3months-base	1.00[0.00,2.25]	2.50[1.25,3.75]	0.00[-3.50,1.00]	1.00[0.50,2.50]	0.209	0.025	0.084	< 0.05*
Δ6months-base	1.00 [0.00,3.00]	2.00[-0.50,5.50]	1.00[0.50,3.00]	1.00[-0.50,2.50]	0.462	0.596	0.519	0.668
CDR								
Δ6months-base	0.00 [0.00,0.00]	-1.00[-1.00,0.00]	0.00 [-1.00,0.50]	0.00 [0.00,0.00]	0.050	0.050	1.000	< 0.05*
GDS-d								
Δ6months-base	-1.00[-3.00,7.00]	-3.00[-5.00,-2.00]	1.00[-2.00,15.50]	6.00[-1.00,18.50]	0.016*	0.034	0.602	< 0.05*
IQ								
Δ6months-base	4.00[-2.00,8.00]	5.00[0.00,10.00]	6.00[1.00,26.00]	-2.00[-9.00,7.50]	0.295	0.753	0.173	0.351
MQ								
Δ6months-base	2.00[-1.00,12.00]	12.00[1.00,16.00]	0.00[-0.50,9.50]	-4.00[-24.50,3.00]	0.075	0.169	0.172	0.103
AQ								
Δ6months-base	3.00[0.00,9.00]	13.00[0.75,29.00]	3.00[3.00,9.00]	-2.00[-7.75,2.25]	0.080	0.714	0.067	0.101

Control group refers to the group that did not receive UCB or EPO therapy

UCB group refers to the group that received only UCB therapy

The UCB + EPO group refers to the group that received both UCB and EPO therapy

Δ refers to the changes of functional assessment scores between post 180 days from therapy and baseline (post 180 days – baseline)

All scores are demonstrated as median [Q1, Q3]

^*a*^*P*-values from Mann-Whitney U test, *P < 0.0167 after Bonferroni correction

^*b*^*P*-values from Kruskal-Wallis test, *P < 0.05

Score of medical research council were summarized values from 22 movements on each side of 8 joints (Shoulder flexion/extension/abductor/adduction, Elbow flexion/extension, Wrist flexion/extension, Finger flexion/extension/abductor/adduction, Hip flexion/extension /abductor/adduction, Knee flexion/extension, Ankle dorsiflexion/plantarflexion, Toe flexion/extension). In the Daniels & Worthingham grade, Zero is 0 points, Trace is 5 points, Poor- is 10 points, Poor is 20 points, Poor + is 30 points, Fair- is 40 points, Fair is 50 points, Fair + is 60 points, and Good- is Scoring was 70 points, Good was 80 points, Good + was 90 points, and Normal was 100 points

*UCB* Umbilical Cord Blood, *EPO* Erythropoietin, *f/u* follow up, *FIM* Functional Independence Measure, *NHISS* National Institutes of Health Stroke Scale, *MMT* Manual Motor Test, *BBS* Berg Balance Scale, *TIS* Trunk Impairment Scale, *MFT* Manual Function Test, *FMA* Fugl-Meyer Assessment, *MMSE* Mini-Mental Status Examination, *MoCA* Montreal Cognitive Assessment, *CDR* Clinical Dementia Rating, *GDS-d* Geriatric Depression Scale, *IQ* Intelligence Quotient, *MQ* Memory Quotient, *AQ* Aphasia Quotient

### Subgroup analysis according to time between stroke onset and the intervention

A subgroup analysis was performed based on the time interval between stroke onset and intervention. The subgroups were divided into < 90-day (*n* = 7) and ≥ 90-day (*n* = 3) groups in the treatment groups (both the UCB and UCB + EPO groups). The FIM motor subscale showed a greater improvement in the < 90-day group than in the ≥ 90-day group (Δbaseline-three months, mean: 13.29 for the 90-day group vs. 1.50 for the ≥ 90-day group, *P* = 0.04; Δbaseline-six months, mean: 17.00 for the 90-day group vs. 6.33 for the ≥ 90-day group, *P* = 0.02). Regarding the FMA of the affected side, the Δbaseline-three months values showed a greater improvement in the 90-day group (17.00 for the 90-day group vs. 6.33 for the ≥ 90-day group, *P* = 0.04).

### Changes in DTI and EEG

Although all patients underwent DTI, only 9 out of the 15 patients (3 patients per group) were available for comparison analysis before and after therapy for technical reasons, including inappropriate head fixation. The quantitative DTI parameters (FA, ADC, fibre number) are summarised in Supplementary Table 5. No statistically significant differences were observed in the DTI parameters before and after therapy (Supplementary Table 5). Upon examining representative patient tractography in each group, the UCB + EPO group showed an apparent but non-significant increase in tract fibres of the CST, SST, cingulum, and AF compared to those in the UCB-only and control groups. However, these findings were observational only, based on small subset of patients, and did not reach statistical significance (Supplementary Fig. 2). Additionally, in the UCB + EPO group, increased crossing of fibres at the loci of the CST that extended to the non-lesioned hemispheres was observed.

Stroke-damaged areas often exhibit increased slow waves, indicating neuronal dysfunction and decreased cerebral blood flow [53]. Therefore, to obtain brain waves, all 15 participants underwent EEG. However, 1 participant from the UCB + EPO group was excluded because of poor data quality, and data from 14 participants were included in the analysis.

A significant reduction in frontal alpha2 power was observed in the UCB + EPO group over six months, indicating increased cortical activation. Similarly, decreases in temporal alpha1 and frontal theta power were also detected. Compared with the UCB group, the UCB + EPO group showed a comparable pattern, with a more pronounced reduction in frontal alpha2 activity. In contrast, the UCB-only group showed minimal changes relative to the control group, suggesting limited effects of UCB monotherapy on EEG power over time. Topographical maps (Supplementary Fig. 3) revealed, that the UCB + EPO group exhibited broader and more prominent decreases in the frontal and temporal regions across the alpha and theta bands compared to the other groups.

### Laboratory evaluation

Peripheral blood samples from all patients were used to assess mRNA levels. qRT-PCR analysis revealed that mRNA levels of IL-1β and IL-8 showed rapid increase potentially due to immune suppression before treatment and gradual decrease following the therapy in the UCB + EPO group without statistical significance (Supplementary Fig. 4).

Cytokine antibody array analysis was performed using available samples from two patients (one in the UCB and one in the UCB + EPO group). In the cytokine antibody array, no significant changes were observed, nevertheless, numerical increases in PDGF-BB, BDNF, MCP-1, FGF-9, and IGFBP-1 were noted in the UCB + EPO group, as detailed in the Supplementary Material (Supplementary Fig. 5, Supplementary Table 6).

## Discussion

This pilot clinical study was designed primarily to evaluate the safety and feasibility of combined UCB + EPO therapy in patients with subacute stroke, extending previous findings in children with cerebral palsy [15] Unlike cerebral palsy, which involves a chronic brain lesion, this study focused on patients with subacute stroke within nine months of stroke onset (median 78 days after stroke onset, [64.00, 116.00]). Most neuroplasticity associated with functional recovery occurs within the first three months after stroke onset during the acute to subacute period [54]. Preclinical and clinical studies suggest that the optimal therapeutic window for cell therapy may be within the first week after stroke [55], although benefits may extend into the subacute phase. In our pilot trial, patients were enrolled between 1 and 9 months after stroke onset [56]. This broader enrolment window enabled us to explore the feasibility and potential effects of UCB + EPO therapy across a wider spectrum, ranging from early to the late subacute stages.

To date, interventional studies investigating functional recovery in patients with subacute stroke have primarily focused on rehabilitation methodologies, such as robotic therapy or non-invasive brain stimulation techniques [57, 58]. Pharmacological approaches have also been studied but have not yielded consistent outcomes [59–62]. Given the limited interventions available for stroke recovery, we considered the potential of cell therapy. However, the optimal therapeutic dose of UCB remains uncertain. Prior studies have suggested potential dose–response relationships [63]. In our study, all patients received at least 2 × 10⁷ TNC/kg, which lies within the lower range of effective doses according to our previous study [24]. The intravenous route was selected in this study because of its feasibility and safety, as reported in previous clinical trials [20, 22, 24]. 

This study aimed to explore the safety and efficacy of UCB, a therapeutic cell source, as well as those of rhEPO co-administration, which is expected to be neurotrophic [16]. Given the authors regarded neuroplasticity as the main therapeutic mechanism for patients with stroke in this critical recovery phase, supportive data that might demonstrate this effect, including MRI and EEG, were also analysed. Although exploratory analyses of functional outcomes were conducted, the small sample size and absence of a priori power calculation indicate that these efficacy findings are hypothesis-generating and should be interpreted with caution.

### Safety

Only one serious AE in the UCB group, hydrocephalus, was reported which was not determined to be related to the therapy, considering the intracerebral and intraventricular haemorrhagic lesions and the onset time at five months after therapy. While there was a total of nine AEs reported in the UCB and UCB + EPO groups, only one event was potentially related to the therapy: chest discomfort occurring the day after the infusion. The patient, who was in a psychologically unstable state (depression) before therapy, showed no abnormalities in vital signs, including oxygen saturation or electrocardiographic findings, during extensive monitoring. The patient’s symptoms resolved without medical treatment, indicating a psychological response. Known side effects of rhEPO, such as thrombosis, nausea, pyrexia, headache, generalised weakness, and superficial phlebitis, were not detected [64, 65]. Additionally, none of the participants in the UCB + EPO group required phlebotomy as their haemoglobin levels did not exceed 13.6 g/dL after EPO administration. Safety monitoring throughout the study included serial physical examinations, laboratory testing, and ECGs during hospitalization and scheduled follow-up visits, and all adverse events and serious adverse events were prospectively recorded and adjudicated. Given these findings, further research involving a larger number of participants should be conducted; though, it seems unlikely that UCB + EPO therapy will cause significant AEs. However, as follow-up in this pilot study was limited to 180 days, longer-term monitoring will be required to fully establish the safety profile. Previous clinical studies have also reported potential detrimental effects of EPO in patients with stroke [66, 67], underscoring the importance of prioritising safety as the primary endpoint in the present pilot study.

### Functional assessment

At six months, the UCB + EPO group showed significant improvements in both FIM total and FIM motor scores, as well as in depressive mood (GDS-d) compared with the control group. The BBS score also increased more in the UCB + EPO group, but this difference was not significant after Bonferroni correction and thus should be interpreted with caution. Exploratory analyses of cognitive function (MMSE, MoCA, and CDR) suggested potential group differences in omnibus testing, although none of the pairwise comparisons remained significant after correction. Therefore, these findings should be regarded as preliminary signals rather than confirmatory evidence. No statistically significant differences were observed between the UCB-only and control groups.

Regarding the synergistic effect of UCB and rhEPO, this research team has conducted various studies on animal models and patients with brain injuries [15, 24, 68], found that rhEPO co-administration was more effective than UCB alone. In a hypoxic-ischaemic encephalopathy model that shares pathological signalling pathways in the cerebral tissue, UCB + rhEPO combination therapy was shown to be more effective, with the downregulation of inflammatory cytokines and a remarkable increase in the phosphorylation of Akt [68]. It potentiated anti-apoptotic responses by decreasing Bax and increasing Bcl-2 expression. In clinical studies on children with cerebral palsy, the combination therapy group showed the highest increase in the primary outcome, Gross Motor Performance Measure score [15, 24]. 

In subgroup analysis, UCB treatment within 90 days of stroke onset was considered more effective. As previously mentioned, the subacute phase—within three months after stroke, is considered a critical period during which neuroplasticity is the most active [54]. Given this context, initiating intensive treatment during the subacute phase of stroke rehabilitation rather than in the chronic phase is likely to be more beneficial.

### Mechanism of therapeutic effect assessments

The influence of transplanted UCB cells on brain tissue regeneration remains unclear. Recent review has highlighted that, due to major anatomical and biological barriers in the adult brain such as the loss of radial glial guidance, glial scar formation, and limited endogenous neurogenesis, true neurorestoration after ischemic stroke is currently considered unachievable. Instead, the beneficial effects of cell therapies are thought to arise mainly from bystander mechanisms such as immunomodulation, trophic support, and facilitation of neuroplasticity leading to functional recovery rather than tissue replacement [69]. 

Previous UCB infusion studies analysing tractography have reported improvements in the CST, posterior thalamic radiata, and other white matter areas [24, 70]. However, the small number of participants in this study limited the ability to detect consistent patterns, and the observed effects remained marginally non-significant. Although not well established, recovery through transcallosal regeneration has been suggested as a possible mechanism for compensatory neuroplasticity [71, 72]. In the UCB + EPO group, increased fibres were observed in the CST, extending to the non-lesioned hemisphere through the corpus callosum, with clinical improvements in muscle strength on both the hemiplegic and non-hemiplegic sides (total MRC scale on the hemiplegic side/on the non-hemiplegic side, baseline 330/1260, six months after therapy 725/1440).

Quantitative EEG can reveal early and subtle neurophysiological changes after stroke, often persisting beyond observable clinical improvement [73, 74]. Delta (1–4 Hz) activity reflects slow brain waves associated with tissue injury, whereas alpha (8–14 Hz) represents cortical idling that decreases with activation, and beta (14–30 Hz) is linked to an active cortical state [75, 76]. In this study, a more pronounced reduction in frontal delta-theta power was observed in the UCB + EPO group compared with the other groups, suggesting enhanced cortical reactivation following therapy in post-stroke neuronal impairments. Elevated IL-8 levels were also observed in this study, aligning with previous UCB and EPO studies, where cytokines increased in the “more-improved” group. IL-8 is known to aid angiogenesis [77], while IL-1β, which typically is pro-inflammatory, has shown neuroprotective characteristics in injured brains [15]. Compared to baseline, a ten-fold increase in these cytokines was observed in the UCB + EPO group on the day of infusion, which was maintained at higher levels than other groups for 30 days. Using ELISA, this study confirmed that PDGF-BB, BDNF, MCP-1, FGF-9, and IGFBP-1 levels were increased in blood samples from the UCB + EPO group compared to those from the UCB group. In addition to BDNF, representative cytokines that promote the growth and differentiation of new neurones and synapses, MCP-1 and IGFBP-1 also play important roles in neural regeneration, providing a reference for the superior therapeutic effects observed in the UCB + EPO group in this study.

### Limitations

This study has some limitations. First, the trial included only a small number of participants in each group, which limited the statistical power and generalisability of the findings. Second, the follow-up period of 180 days may have been too short to capture the full course of recovery after subacute stroke. Third, although there was no statistically significant difference in post-stroke duration among the groups, the control group had the longest mean duration, partly because one participant received the intervention 237 days post-stroke. However, sensitivity analyses that excluded these individual yielded similar results, suggesting a minimal impact on the overall findings. Fourth, the quality of some biological samples limited the cytokine array and DTI analyses, thereby reducing the robustness of these mechanistic findings. Fifth, detailed immunophenotyping of the infused UCB units (e.g., CD34 + and, CD133 + subpopulations) could not be performed; only TNC counts, viability, and HLA matching were available. Sixth, the EEG results were only available at the group level, because the analysis platform did not allow the extraction of individual raw data values. Finally, the use of cryopreserved UCB units may have influenced the cellular composition and therapeutic potential, as cryopreservation has been reported to reduce the viability of certain subpopulations, including CD34 + progenitor cells [78, 79].

Therefore, future trials should be designed with larger sample sizes, longer follow-up period, comprehensive cell characterisation, and, if possible, comparisons between fresh and cryopreserved UCB cells to strengthen the evidence base for UCB + EPO therapy in subacute stroke.

## Conclusion

This study is the first to simultaneously investigate the safety and efficacy of UCB and EPO in patients with subacute stroke. With regard to safety, the intervention was deemed to have a low risk of AEs. In terms of efficacy, the study examined not only the functional outcomes of patients but also changes at the molecular level, as well as alterations in brain imaging and biosignals. This comprehensive approach allowed for a detailed understanding of the favourable therapeutic effects observed among patients, supporting the potential efficacy and safety of UCB + EPO combination therapy in subacute stroke. Further research with larger sample sizes and extended follow-up periods is required to validate our findings.

## Supplementary Information

Supplementary material 1.

Supplementary material 2.

Supplementary material 3.

Supplementary material 4.

Supplementary material 5.

Supplementary material 6.

Supplementary material 7.

Supplementary material 8.

Supplementary material 9.

Supplementary material 10.

Supplementary material 11.

Supplementary material 12.

## Data Availability

The datasets generated and/or analysed during the current study are not publicly available due to institutional policy, but de-identified data may be obtained from the corresponding author upon reasonable request and with appropriate ethical approval.
