# Supplementary material for: Safety and efficacy of allogeneic umbilical cord blood cells and erythropoietin combination therapy in patients with subacute stroke
Source: Stem Cell Res Ther. 2025 Dec 27;17:56. doi: 10.1186/s13287-025-04856-8 (PMC12853616; doi:10.1186/s13287-025-04856-8)
Supplement: Supplementary file 1 — Supplementary material 1. [file 13287_2025_4856_MOESM1_ESM.docx]

Supplementary Figure 1. The cooperation of investigators in the study institute to maintain double-blind control


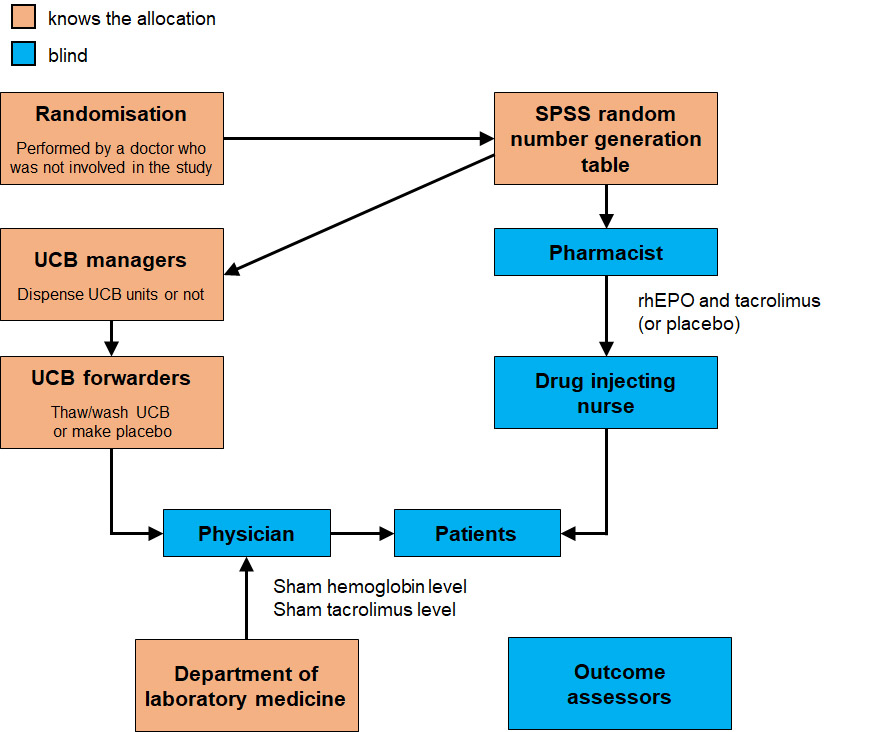


Randomisation was conducted by an independent researcher not involved in patient care. The randomisation code was delivered only to the pharmacist, who prepared UCB/EPO or matched placebo in indistinguishable packaging labeled with serial numbers. Nurses administered the intervention according to the serial number without knowledge of group allocation. Treating physicians, outcome assessors, and patients remained blinded throughout the study.
