# Supplementary material for: Safety and efficacy of allogeneic umbilical cord blood cells and erythropoietin combination therapy in patients with subacute stroke
Source: Stem Cell Res Ther. 2025 Dec 27;17:56. doi: 10.1186/s13287-025-04856-8 (PMC12853616; doi:10.1186/s13287-025-04856-8)
Supplement: Supplementary file 2 — Supplementary material 2. [file 13287_2025_4856_MOESM2_ESM.docx]

Supplementary Figure 2. Tractography of the most typical subject UCB+EPO, UCB, and Control group.


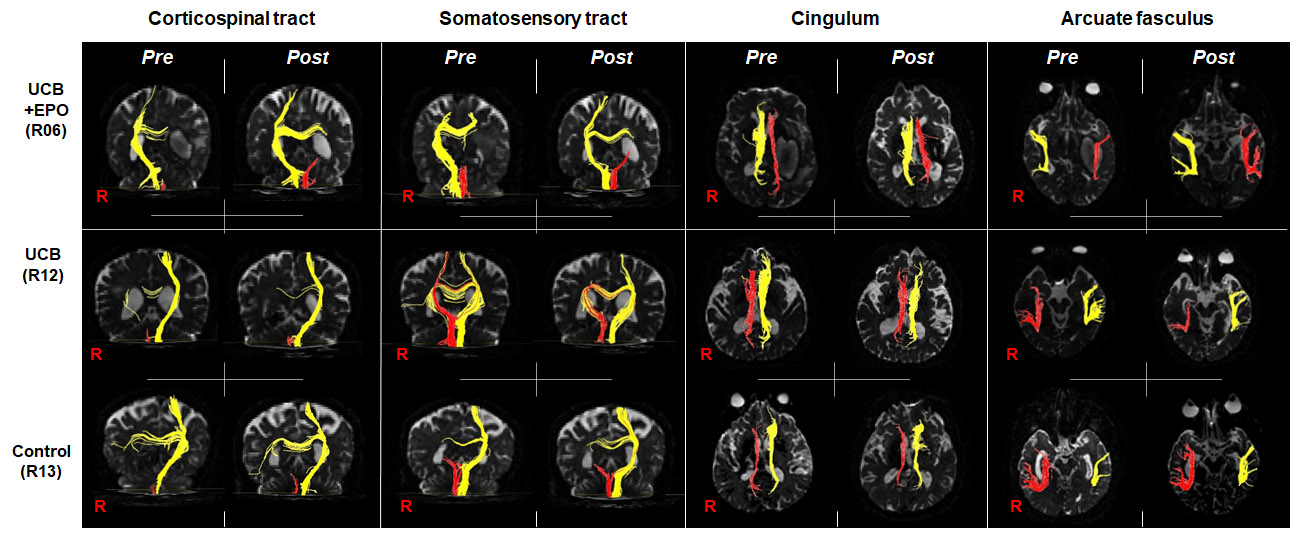


Analysis was performed on the corticospinal tract, somatosensory tract, cingulum, and arcuate fasciculus, and was confirmed before and 6 months after the therapy. The red tract signifies the affected side, while the yellow tract indicates the unaffected side.

UCB, Umbilical Cord Blood; EPO, Erythropoietin
