# Supplementary material for: Safety and efficacy of allogeneic umbilical cord blood cells and erythropoietin combination therapy in patients with subacute stroke
Source: Stem Cell Res Ther. 2025 Dec 27;17:56. doi: 10.1186/s13287-025-04856-8 (PMC12853616; doi:10.1186/s13287-025-04856-8)
Supplement: Supplementary file 3 — Supplementary material 3. [file 13287_2025_4856_MOESM3_ESM.docx]

Supplementary Figure 3. Electroencephalography (EEG) changes pre- and post- therapy in each group.


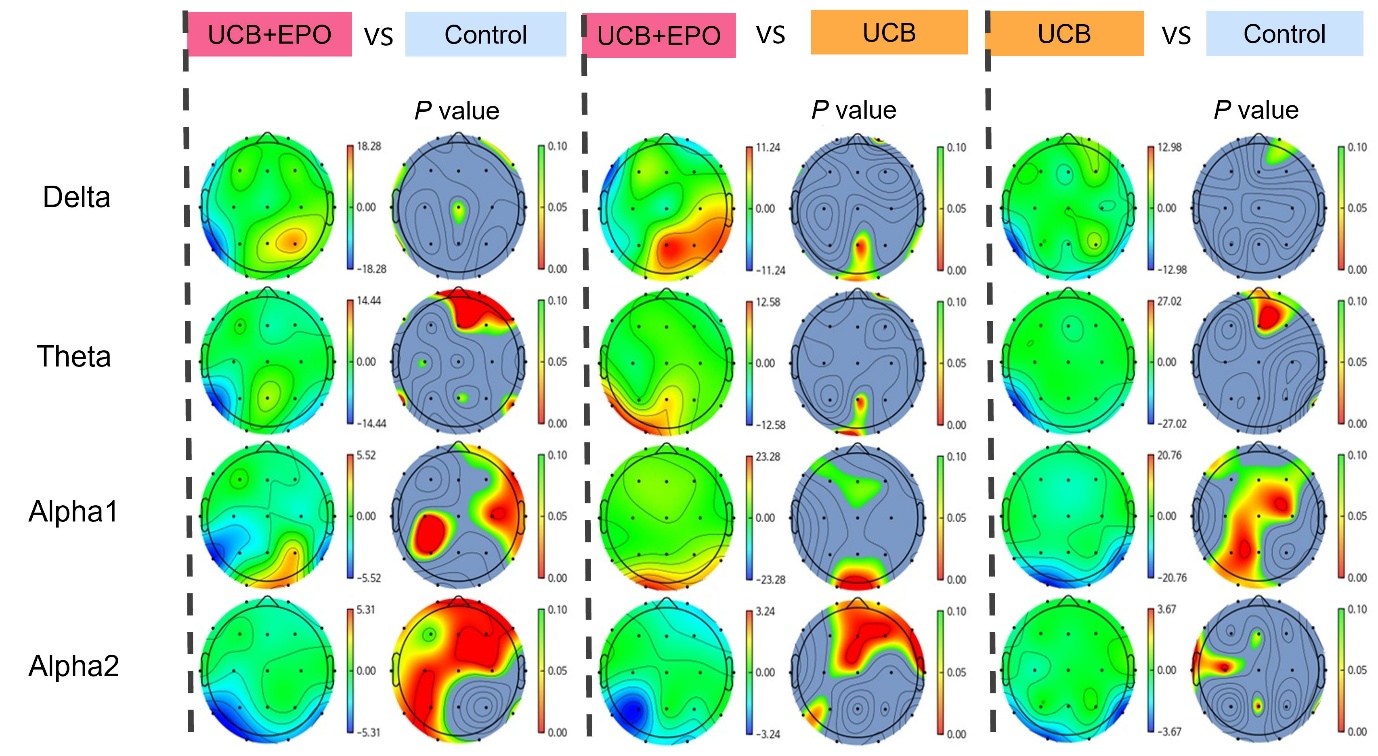


All data represent the difference between baseline and 180 days after therapy. Columns 1, 3, and 5 display the differences in change scores (pre- vs. post-therapy) between groups, and Columns 2, 4, and 6 show the p-values for these differences. The differences for each group are displayed according to the EEG waves aligned with the 10-20 system.

UCB, Umbilical Cord Blood; EPO, Erythropoietin
