# Supplementary material for: Safety and efficacy of allogeneic umbilical cord blood cells and erythropoietin combination therapy in patients with subacute stroke
Source: Stem Cell Res Ther. 2025 Dec 27;17:56. doi: 10.1186/s13287-025-04856-8 (PMC12853616; doi:10.1186/s13287-025-04856-8)
Supplement: Supplementary file 4 — Supplementary material 4. [file 13287_2025_4856_MOESM4_ESM.docx]

Supplementary Figure 4. Validation of the changes in cytokines over time before and after therapy in UCB+EPO, UCB, and control group.


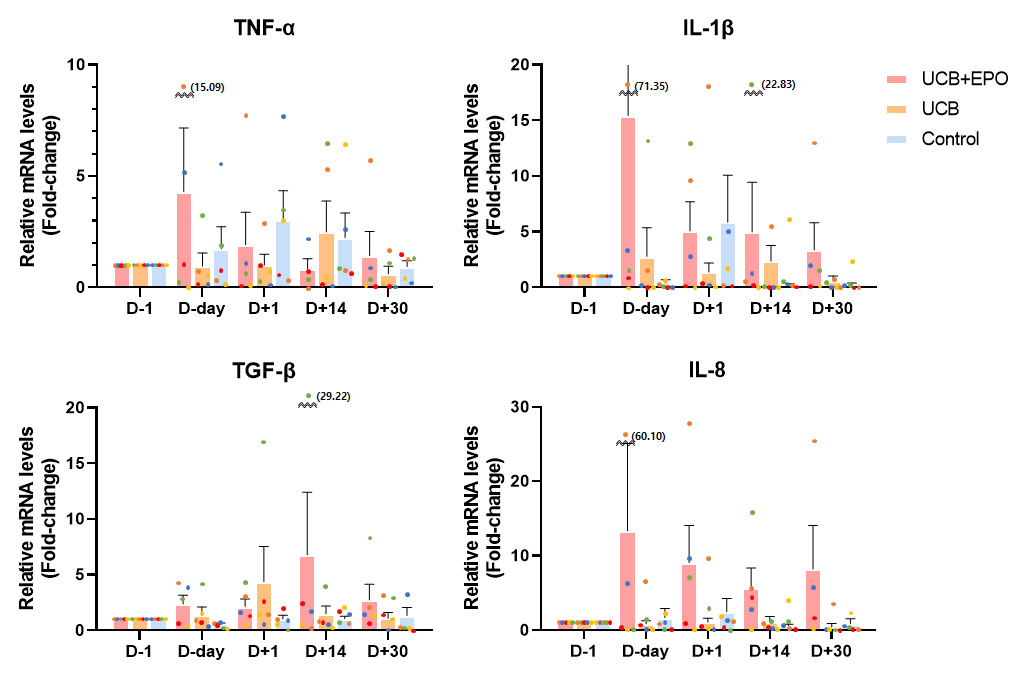


Each colored dot represents an individual patient, and the same color is used consistently across panels to indicate the same patient (consistent with Figure 3). Bars indicate the group mean, and error bars represent standard deviation. Quantitative real-time PCR for inflammation-related cytokines (TNF-α, TGF-β, IL-1β, and IL-8) was analyzed in duplicate for each group (n=5 per group).

D-1: One day before therapy, D-day: The day of therapy, D+1: One day after therapy, D+14: 14 days after therapy, D+30: 30 days after therapy

UCB, Umbilical Cord Blood; EPO, Erythropoietin
