# Supplementary material for: Safety and efficacy of allogeneic umbilical cord blood cells and erythropoietin combination therapy in patients with subacute stroke
Source: Stem Cell Res Ther. 2025 Dec 27;17:56. doi: 10.1186/s13287-025-04856-8 (PMC12853616; doi:10.1186/s13287-025-04856-8)
Supplement: Supplementary file 5 — Supplementary material 5. [file 13287_2025_4856_MOESM5_ESM.docx]

Supplementary Figure 5. The cytokine antibody array before and after therapy in UCB+EPO and UCB


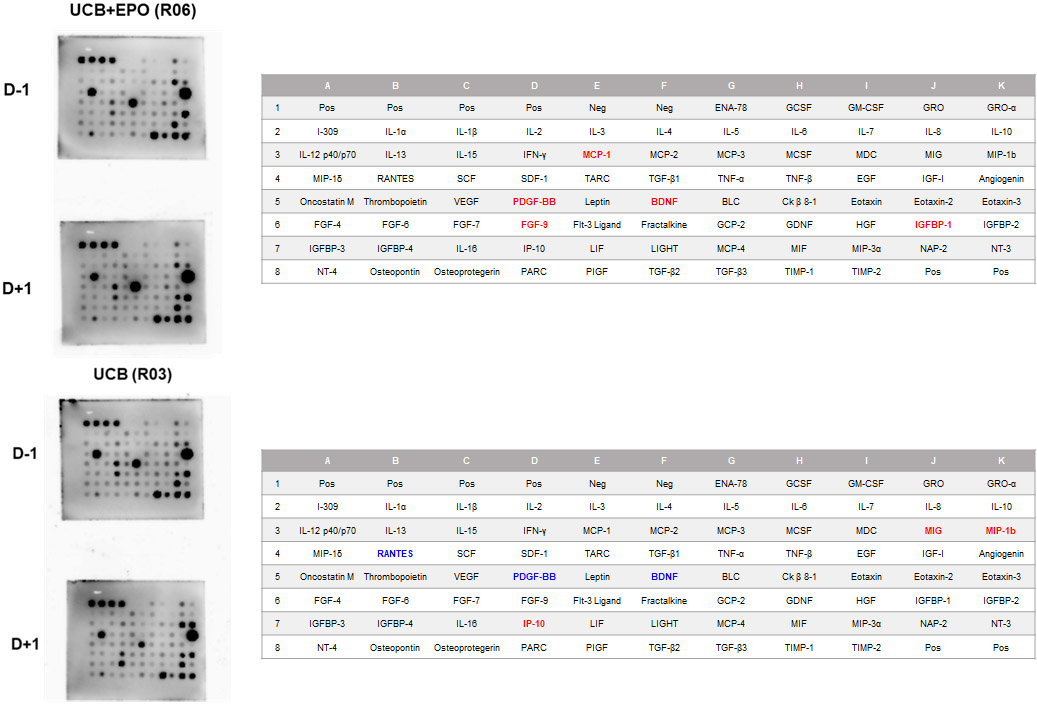


The Cytokine antibody array was performed by the Human Cytokine Antibody Array C5 (RayBiotech, Inc) for semi quantitative detection of 80 proteins according to manufacturer’s instructions. (each groups n=1). The red-labeled markers indicate dots with increased expression, while blue-labeled markers indicate dots with decreased expression. In UCB+EPO participant (R06), PDGF-BB, BDNF, MCP-1, FGF-9 and IGFBP-1 to increase in the UCB+EPO showed a tendency to increase. MIG, MIP-1β, IGFBP-1, and IP-10 increased in the UCB participant (R03), though none were statistically significant.

UCB, Umbilical Cord Blood; EPO, Erythropoietin; Pos: Positive; Neg, Negative; ENA-78, Epithelial Neutrophil-Activating Peptide 78; GCSF, Granulocyte Colony-Stimulating Factor; GM-CSF, Granulocyte-Macrophage Colony-Stimulating Factor; GRO, Growth-Regulated Oncogene; GRO-α, Growth-Regulated Oncogene Alpha; I-309, T cell-derived chemokine (CCL1); IL-1α, Interleukin 1 Alpha; IL-1β, Interleukin 1 Beta; IL-2, Interleukin 2; IL-3, Interleukin 3; IL-4, Interleukin 4; IL-5, Interleukin 5; L-6, Interleukin 6; IL-7, Interleukin 7; IL-8, Interleukin 8; IL-10, Interleukin 10; IL-12, Interleukin 12; IL-13, Interleukin 13; IL-15, Interleukin 15; IFN-γ, Interferon Gamma; MCP-1, Monocyte Chemoattractant Protein-1; MCP-2, Monocyte Chemoattractant Protein-2; MCP-3, Monocyte Chemoattractant Protein-3; MCSF, Macrophage Colony-Stimulating Factor; MDC, Macrophage-Derived Chemokine; MIG, Monokine Induced by Gamma Interferon; MIP-1b, Macrophage Inflammatory Protein 1 Beta;; MIP-1δ, Macrophage Inflammatory Protein 1 Delta; RANTES, Regulated upon Activation, Normal T Cell Expressed and Secreted; SCF, Stem Cell Factor; SDF-1, Stromal Cell-Derived Factor 1; TARC, Thymus and Activation-Regulated Chemokine; TGF-β1, Transforming Growth Factor Beta 1; TNF-α, Tumor Necrosis Factor Alpha; TNF-β, Tumor Necrosis Factor Beta; EGF, Epidermal Growth Factor; IGF-I, Insulin-like Growth Factor I; VEGF, Vascular Endothelial Growth Factor; PDGF-BB, Platelet-Derived Growth Factor BB; BDNF, Brain-Derived Neurotrophic Factor; BLC, B Lymphocyte Chemoattractant; Ck β 8-1, Chemokine Beta 8-1; FGF-4, Fibroblast Growth Factor 4; FGF-6, ; Fibroblast Growth Factor 6; FGF-7, Fibroblast Growth Factor 7; FGF-9, Fibroblast Growth Factor 9; Flt-3 Ligand, Fms-like Tyrosine Kinase 3 Ligand; GCP-2, Granulocyte Chemotactic Protein 2; GDNF, Glial Cell Line-Derived Neurotrophic Factor; HGF, Hepatocyte Growth
