# Supplementary material for: Safety and efficacy of allogeneic umbilical cord blood cells and erythropoietin combination therapy in patients with subacute stroke
Source: Stem Cell Res Ther. 2025 Dec 27;17:56. doi: 10.1186/s13287-025-04856-8 (PMC12853616; doi:10.1186/s13287-025-04856-8)
Supplement: Supplementary file 6 — Supplementary material 6. [file 13287_2025_4856_MOESM6_ESM.docx]

**Supplementary Methods**

**Inclusion Criteria**

- Adult patients aged 20 years and older, both male and female.

- Patients with unilateral brain lesion and definite hemiplegia involving upper extremity paralysis

- Patients who have had a stroke for more than 30 days but less than 9 months.

- unilateral supratentorial lesion of infarct or hemorrhage

- Those who have heard a detailed explanation of this clinical trial, fully understood it, and voluntarily decided to participate after giving written consent by themselves or their legal representatives.

**Exclusion Criteria**

- Uncontrolled hypertension

- Patients with impaired capacity to provide informed consent without a legally authorized representative

- Female who are pregnant or not avoiding pregnancy

- Abnormal blood laboratory data, such as ALT/AST > 120IU/L, serum creatine > 1.8mg/dL, total bilirubin > 1.8mg/dL, total WBC count < 3000/mm^3^, hemoglobin > 16g/dL, and platelet count < 150,000/uL or > 675,000/uL

- Clinically significant infection such as pneumonia, pyelonephritis, and sepsis during the screening period

- Medical instability due to serious cardiovascular, gastrointestinal, pulmonary, or endocrinological disorder

- All kinds of immune deficiency by innate or acquired causes

- Diagnosed malignant tumor except for complete remission more than ten years

- Having side effects to medications used in the study; EPO and tacrolimus

- Any other condition that would pose an unacceptable risk to the patient’s safety or interfere with trial assessments, as determined by the study team

**Erythropoietin administration precautions**

- Patients with hypersensitivity to erythropoietin preparations

- Patients hypersensitive to mammalian cell-derived drugs or human albumin

- Patients with a history of seizures

**Tacrolimus administration precautions**

- Patients with hypersensitivity to tacrolimus or macrolide compounds

- Patients taking cyclosporine or bosentan

- Patients taking potassium-sparing diuretics

**Laboratory Evaluation**

The whole blood collected into EDTA-anticoagulated tubes was centrifuged for 20 min at approximately 400 × g (2000 rpm) without brake. Plasma in the separated upper layer was transferred into a new tube and centrifuged for 10 min at approximately 12,000 × g (10000 rpm) to remove debris. After adding EDTA-PBS to the remaining blood, PBMCs were separated by density gradient centrifugation for 20 min at approximately 400 × g (2000 rpm) using Ficoll (GE Healthcare, USA). The white and cloudy layer containing PBMCs was gently transferred into a new tube, washed with PBS, and the pelleted cells were stored in a deep freezer.

**Reverse transcription polymerase chain reaction (RT-PCR)**

To extract total RNA from PMSCs, we used to TRIzol (Invitrogen, Carlsbad, CA, USA) following the manufacturer’s protocol. cDNA was generated from total RNA from PBMCs using ReverTra RT Master Mix kit (Toyobo Co., Osaka, Japan). The relative expression of inflammation marker was confirmed by real-time PCR using SYBR Green Mix (2x) (Promega, Madison, WI, USA). We designed specific primers; TNF-α forward, 5’-CTCTTCTGCCTGCTGCACTTTG-3’, TNF-α reverse, 5’-ATGGGCTACAGGCTTGTCACTC-3’, and IL-1β forward, 5’-TTCTTCGACACATGGGATAACG-3’, IL-1β reverse, 5’-TGGAGAACACCACTTGTTGCT-3’, and TGF-b forward, 5’-CTCGCCAGAGTGGTTATCTT-3’, TGF-b reverse, 5’- AGTGTGTTATCCCTGCTGTCA-3’, and IL-8 forward, 5’-ACTGAGAGTGATTGAGAGTGGAC-3’, IL-8 reverse, 5’- AACCCTCTGCACCCAGTTTTC-3’, and 18s rRNA forward, 5’-GTAACCCGTTGAACCCCATT-3’, 18s rRNA reverse, 5’- CCATCCAATCGGTAGTAGCG-3’. Duplex qPCR reactions were performed for each independent sample with ribosomal 18S rRNA as the internal standard. And each sample was performed an equivalent of 100ng mRNA per reaction in duplicate followed by 95℃ for 30s and 60℃ for 30s that cycle was repeated for 35.

**Enzyme-linked immunosorbent assay**

To screen for the secretion of cytokine by therapeutic effect in patient's plasma, Human Cytokine Antibody Array (Abcam, Cambridge, UK, Ab133998) was used and analyzed. For analysis of cytokine array, plasma samples were diluted with phosphate-buffered solution (PBS) according to the manufacturer’s instructions. Block membranes by incubating with 2 mL 1× Blocking Buffer at room temperature (RT) for 30 min. diluted or undiluted sample into each well and incubate for 1.5 - 2 hours at RT. Briefly, the membranes were blocked by incubating with 2 mL 1× Blocking Buffer at RT for 30 min. And diluted plasma sample into each well and incubate for overnight at 4°C. After the overnight incubation, membranes washed 5 time using wash buffer 1, 2 and incubate for 2 hours 1× with Biotin-Conjugated Anti-Cytokines. Next, 1× HRP-Conjugated Streptavidin into each well incubated for 2 hours at RT. Thereafter, washed membranes were detected by chemiluminescence.

**Brain imaging procedure**

As for DTI, DTI imaging parameters were set as follows: acquisition matrix = 128 × 128; field of view = 240mm × 240mm; repetition time = 13,000 ms; echo time = 109.1 ms; parallel imaging reduction factor = 2.0; b = 1000 s/mm2; number of excitations = 2; and slice thickness = 2 mm with 3.0 Tesla GE Signa System (General Electric, Milwaukee, WI, USA). Affine multi-scale two-dimensional registration at the Oxford Centre for Functional Magnetic Resonance Imaging of Brain (FMRIB) Software Library (FSL; www.fmrib.ox.ac.uk/fsl) was used for removal of eddy current-induced image distortions. DTI-Studio software (CMRM, Johns Hopkins Medical Institute, Baltimore, MD, USA) was used to reconstruct the neural tracts. Four neural tracts were reconstructed as follows. 1) Corticospinal tract (CST): two ROIs were placed on the isolated CST area: ROI 1 - anterior portion of the upper pons on the axial image, and ROI 2 - anterior portion of the lower pons on the axial image. 2) Somatosensory tract (SST), ROI 1 - posterior portion of the upper pons on the axial image, and ROI 2 - posterior portion of the lower pons on the axial image: 3) For the cingulum, ROI 1 – anterior portion of the cingulum on the coronal image, and ROI 2 – posterior portion of the cingulum on the coronal image. 4) For the arcuate fasciculus (AF), ROI 1 - the deep white matter of the posterior parietal portion of the SLF on the axial image, and ROI 2 - the posterior temporal lobe on the axial image. The fractional anisotropy (FA), apparent diffusion coefficient (ADC) values and fibre number (FN) were measured using DTI-Studio software.
