# Supplementary material for: Safety and efficacy of allogeneic umbilical cord blood cells and erythropoietin combination therapy in patients with subacute stroke
Source: Stem Cell Res Ther. 2025 Dec 27;17:56. doi: 10.1186/s13287-025-04856-8 (PMC12853616; doi:10.1186/s13287-025-04856-8)
Supplement: Supplementary file 7 — Supplementary material 7. [file 13287_2025_4856_MOESM7_ESM.docx]

Supplementary Table 1. Comparison of manual muscle test (MMT) grade.

| Daniels & Worthingham | Kendall & McCreary | Scoring used in this study | Explanation |
| --- | --- | --- | --- |
| Normal (N) | 100 % | 100 | Hold test position against maximal resistance |
| Good+ (G+) |  | 90 | Holds test position against moderate to strong pressure |
| Good (G) | 80% | 80 | Holds test position against moderate resistance |
| Good- (G-) |  | 70 | Holds test position against slight to moderate pressure |
| Fair+ (F+) |  | 60 | Holds test position against slight resistance |
| Fair (F) | 50% | 50 | Holds test position against gravity |
| Fair- (F-) |  | 40 | Gradual release from test position |
| Poor+ (P+) |  | 30 | Moves through partial ROM against gravity or moves through complete ROM gravity eliminated and holds against pressure |
| Poor (P) | 20% | 20 | Able to move through full ROM gravity eliminated |
| Poor- (P-) |  | 10 | Moves through partial ROM gravity eliminated |
| Trace (T) | 5% | 5 | No visible movement; palpable or observable tendon prominence / flicker contraction |
| Zero (Z) | 0% | 0 | No palpable or observable muscle contraction |

Score of medical research council were summated values from 22 movements on each side of 8 joints (Shoulder flexion/extension/abductor/adduction, Elbow flexion/extension, Wrist flexion/extension, Finger flexion/extension/abductor/adduction, Hip flexion/extension/abductor/adduction, Knee flexion/extension, Ankle dorsiflexion/plantarflexion, Toe flexion/extension).

MMT, Manual Muscle Test; ROM, Range of Motion
