# Supplementary material for: Safety and efficacy of allogeneic umbilical cord blood cells and erythropoietin combination therapy in patients with subacute stroke
Source: Stem Cell Res Ther. 2025 Dec 27;17:56. doi: 10.1186/s13287-025-04856-8 (PMC12853616; doi:10.1186/s13287-025-04856-8)
Supplement: Supplementary file 8 — Supplementary material 8. [file 13287_2025_4856_MOESM8_ESM.docx]

Supplementary Table 2. Brain Magnetic Resonance Imaging.

| Group | Patient number | Onset to MRI/Onset to Intervention (days) | Brain MRI* | MRI Finding | Baseline FAC/ FIM total score |
| --- | --- | --- | --- | --- | --- |
| UCB+EPO group | R09 | 11/71 | 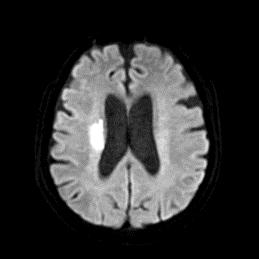 | Cerebral infarction in Rt. periventricular WM, corona radiata to BG | 2/89 |
|  | R06* | 21/80 | 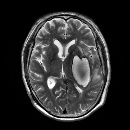 | Subacute hemorrhage in Lt. periventricular WM - BG - temporal lobe | 1/60 |
|  | R09 | 1/64 | 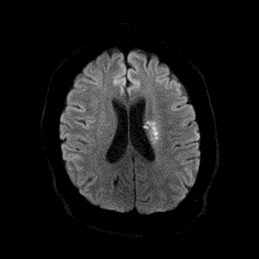 | Acute small infarct in Lt. BG | 4/116 |
|  | R14 | 101/103 | 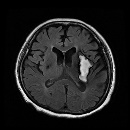 | Subacute ICH in Lt. BG and centrum semiovale. | 0/79 |
|  | R15 | 56/59 | 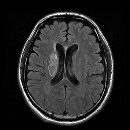 | Acute infarction in the Rt. corpus callosum, recent infarction in the Rt. BG and deep WM | 1/51 |
| UCB group | R01 | 35/116 | 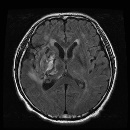 | Occlusion in Rt. MCA proximal M1, Subacute hematoma in Rt. BG | 1/41 |
|  | R03 | 3/90 | 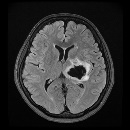 | Acute ICH in Lt. thalamus and posterior limb of IC | 0/73 |
|  | R04* | 50/78 | 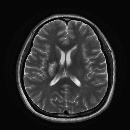 | Subacute to chronic striatocapsular infarction in Rt. BG | 2/83 |
|  | R12* | 47/64 | 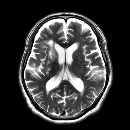 | Wide central WM old infarcts in Rt. hemisphere,  several old lacunar infarcts in Rt. BG | 1/82 |
|  | R16 | 43/54 | 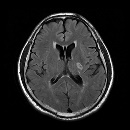 | Subacute to chronic infarction in Lt. posterior limb of IC - BG | 2/88 |
| Control group | R02 | 20/237 | 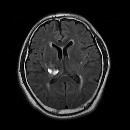 | Subacute hemorrhage in Rt. BG, Thalamus, Multiple old lacunar infarction in Lt. periventricular WM, bilateral BG, and Lt. pons | 3/87 |
|  | R07 | 29/71 | 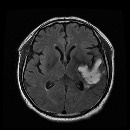 | Subacute to chronic hemorrhage in BG - temporal lobe  - SAH in Rt. parietal sulci, IVH | 0/34 |
|  | R08 | 116/122 | 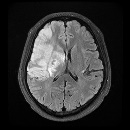 | Acute - subacute infarction with hemorrhagic transformation in Rt. frontotemporal lobes – Rt. BG | 1/95 |
|  | R10 | 10/67 | 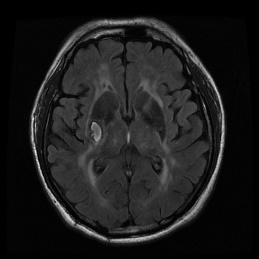 | Approximately 1.7 cm subacute ICH in Rt. basal ganglia, Several old lacunar infarctions in bilateral periventricular WM and Lt. thalamus Several microbleeds | 1/43 |
|  | R13* | 101/138 | 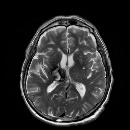 | Old ICH in Rt. thalamus and IC posterior limb | 2/86 |

MRI was selected as the most visible image of the lesion among T2 flair imaging and diffusion tensor imaging.

* Diffusion tensor imaging exist

MRI, Magnetic Resonance Imaging; DTI, Diffusion Tensor Imaging; FA, Fractional Anisotropy; Rt, Right; BG, Basal Ganglia; Lt, Left; WM, white matter; SAH, Subarachnoid Hemorrhage; IVH, Intraventricular Hemorrhage; ICH, Intracerebral Hemorrhage; IC, Internal Capsule; MCA, Middle Cerebral Artery
