# Supplementary material for: Safety and efficacy of allogeneic umbilical cord blood cells and erythropoietin combination therapy in patients with subacute stroke
Source: Stem Cell Res Ther. 2025 Dec 27;17:56. doi: 10.1186/s13287-025-04856-8 (PMC12853616; doi:10.1186/s13287-025-04856-8)
Supplement: Supplementary file 9 — Supplementary material 9. [file 13287_2025_4856_MOESM9_ESM.docx]

Supplementary Table 3. Composition of unrelated allogeneic umbilical cord blood units

| Group | Patient number | Body weight (kg) | Number of TNC  (×10^8^) | TNC per kg (×10^7^) | Viability  (%) | Blood type of units | Number of HLA mismatch† |
| --- | --- | --- | --- | --- | --- | --- | --- |
| UCB+ EPO | R05 (AB+) | 49.0 | 6.35  4.91 | 1.3  1.0 | 96.0  100.0 | A+  B+ | 2  1 |
|  | Total |  |  | 2.3 |  |  |  |
|  | R06 (AB+) | 71.0 | 6.22  4.49  4.90 | 0.9  0.6  0.7 | 100.0  96.0  98.0 | AB+  AB+  AB+ | 2  2  3 |
|  | Total |  |  | 2.2 |  |  |  |
|  | R09  (A+) | 74.6 | 6.74  4.12  7.00 | 0.9  0.6  0.9 | 99.0  100.0  98.0 | A+  A+  A+ | 3  3  3 |
|  | Total |  |  | 2.4 |  |  |  |
|  | R13  (O+) | 75.0 | 5.48  4.25  5.79 | 0.7  0.6  0.8 | 88.0  92.0  89.0 | O+  O+  O+ | 1  2  2 |
|  | Total |  |  | 2.1 |  |  |  |
|  | R15  (A+) | 67.6 | 4.65  4.52  5.24 | 0.7  0.7  0.8 | 98.0  96.0  99.0 | A+  A+  A+ | 2  2  2 |
|  | Total |  |  | 2.2 |  |  |  |
| UCB | R01  (B+) | 65.5 | 4.15  6.51  5.46 | 0.6  1.0  0.8 | 95.0  95.0  96.0 | B+  B+  B+ | 2  3  3 |
|  | Total |  |  | 2.4 |  |  |  |
|  | 7 (A+) | 52.1 | 15.69 | 3.0 | 95.5 | A+ | 2 |
|  | R04  (A+) | 59.8 | 5.37  7.96 | 0.9  1.3 | 97.0  98.0 | A+  A+ | 2  2 |
|  | Total |  |  | 2.2 |  |  |  |
|  | R12  (A+) | 68.0 | 5.02  5.38  4.37 | 0.7  0.8  0.6 | 97.0  99.0  97.0 | A+  A+  A+ | 2  2  2 |
|  | Total |  |  | 2.1 |  |  |  |
|  | R16  (AB+) | 65.8 | 5.67  3.88  4.06 | 0.9  0.6  0.6 | 96.5  95.0  97.0 | AB+  AB+  AB+ | 2  2  2 |
|  | Total |  |  | 2.1 |  |  |  |
| Control | R02  (AB+) | 74.8 | - | - | - | - | - |
|  | R07  (B+) | 55.2 | - | - | - | - | - |
|  | R08  (A+) | 70.9 | - | - | - | - | - |
|  | R10  (AB+) | 70.5 | - | - | - | - | - |
|  | R13  (B+) | 51.0 | - | - | - | - | - |

†UCB unit matched for four of six HLA types A, B, and DRB1 antigens at least with high resolution. 1 and 2 represent one and two mismatched antigens from six HLA antigens respectively.

TNC, Total Nucleated Cell; UCB, Umbilical Cord Blood; HLA, Human Leukocyte Antigen
