# Supplementary material for: Safety and efficacy of allogeneic umbilical cord blood cells and erythropoietin combination therapy in patients with subacute stroke
Source: Stem Cell Res Ther. 2025 Dec 27;17:56. doi: 10.1186/s13287-025-04856-8 (PMC12853616; doi:10.1186/s13287-025-04856-8)
Supplement: Supplementary file 10 — Supplementary material 10. [file 13287_2025_4856_MOESM10_ESM.docx]

Supplementary Table 4. *P*-values from RM-ANOVA in functional assessments

| Variables | *P*-value |
| --- | --- |
|  |  |
| FIM total | 0.86 |
| FIM motor | 0.85 |
| NIHSS | 0.64 |
| MMT affected side | 0.81 |
| BBS | 0.92 |
| TIS | 0.75 |
| MFT affected side | 0.37 |
| FMA affected side | 0.47 |
| MMSE | 0.87 |
| MoCA | 0.58 |

Table S4 shows the p-values from repeated measures ANOVA comparing time-by-group interaction effects in functional assessments among the UCB+EPO, UCB, and Control groups.

FIM, Functional Independence Measure; NIHSS, National Institutes of Health Stroke Scale; MMT, Manual Muscle Testing; BBS, Berg Balance Scale;
TIS, Trunk Impairment Scale; MFT, Manual Function Test; FMA, Fugl-Meyer Assessment; MMSE, Mini-Mental State Examination; MoCA, Montreal Cognitive Assessment.
