# Supplementary material for: Safety and efficacy of allogeneic umbilical cord blood cells and erythropoietin combination therapy in patients with subacute stroke
Source: Stem Cell Res Ther. 2025 Dec 27;17:56. doi: 10.1186/s13287-025-04856-8 (PMC12853616; doi:10.1186/s13287-025-04856-8)
Supplement: Supplementary file 11 — Supplementary material 11. [file 13287_2025_4856_MOESM11_ESM.docx]

|  |  | UCB+EPO group (n=3) | | | | | |  | UCB group (n=3) | | | | | |  | Control group (n=3) | | | | | |
| --- | --- | --- | --- | --- | --- | --- | --- | --- | --- | --- | --- | --- | --- | --- | --- | --- | --- | --- | --- | --- | --- |
|  |  | Affected side | | | Unaffected side | | |  | Affected side | | | Unaffected side | | |  | Affected side | | | Unaffected side | | |
|  |  | FA | ADC | FN | FA | ADC | FN |  | FA | ADC | FN | FA | ADC | FN |  | FA | ADC | TV | FA | ADC | FN |
| CST | Pre | 0.52  (0.05) | 0.72  (0.04) | 91.67  (88.79) | 0.63  (0.01) | 0.72  (0.07) | 1654.00  (132.89) |  | 0.53  (0.07) | 0.76  (0.05) | 352.00  (528.52) | 0.65  (0.03) | 0.75  (0.01) | 1293.00  (85.14) |  | 0.51  (0.07) | 0.78  (0.06) | 220.33  (262.39) | 0.62  (0.03) | 0.82  (0.03) | 1440.33  (429.72) |
|  | Post | 0.52  (0.03) | 0.84  (0.11) | 125.67  (168.69) | 0.68  (0.09) | 0.73  (0.06) | 1463.33  (533.65) |  | 0.55  (0.01) | 0.76  (0.11) | 256.00  (358.05) | 0.65  (0.06) | 0.77  (0.02) | 1193.00  (148.58) |  | 0.49  (0.12) | 0.96  (0.09) | 241.00  (221.98) | 0.63  (0.01) | 0.83  (0.07) | 1316.33  (504.49) |
| SST | Pre | 0.62  (0.02) | 0.76  (0.04) | 540.00  (320.29) | 0.61  (0.01) | 0.77  (0.03) | 1652.00  (337.68) |  | 0.62  (0.02) | 0.73  (0.03) | 916.00  (495.93) | 0.62  (0.03) | 0.74  (0.05) | 1909.00  (253.15) |  | 0.60  (0.01) | 0.77  (0.08) | 525.33  (241.43) | 0.60  (0.01) | 0.78  (0.03) | 1311.67  (648.84) |
|  | Post | 0.59  (0.03) | 0.74  (0.02) | 697.00  (524.35) | 0.61  (0.02) | 0.75  (0.01) | 1780.00  (457.58) |  | 0.65  (0.08) | 0.81  (0.01) | 811.33  (478.43) | 0.64  (0.05) | 0.80  (0.03) | 2139.67  (283.66) |  | 0.63  (0.08) | 0.80  (0.04) | 680.00  (619.15) | 0.63  (0.05) | 0.83  (0.06) | 1385.33  (759.86) |
| Cingulum | Pre | 0.49  (0.09) | 0.86  (0.09) | 470.67  (116.84) | 0.46  (0.02) | 0.87  (0.05) | 854.33  (421.55) |  | 0.52  (0.05) | 0.79  (0.03) | 903.67  (259.23) | 0.55  (0.02) | 0.76  (0.03) | 1230.00  (129.55) |  | 0.46  (0.05) | 0.90  (0.10) | 630.00  (304.87) | 0.50  (0.05) | 0.89  (0.08) | 822.00  (188.04) |
|  | Post | 0.45  (0.06) | 0.90  (0.06) | 465.67  (113.54) | 0.46  (0.04) | 0.83  (0.05) | 834.00  (452.83) |  | 0.54  (0.08) | 0.84  (0.05) | 967.33  (612.83) | 0.57  (0.08) | 0.82  (0.06) | 856.67  (287.01) |  | 0.46  (0.06) | 0.98  (0.06) | 610.67  (357.09) | 0.52  (0.06) | 0.91  (0.10) | 781.33  (154.64) |
| AF | Pre | 0.47  (0.04) | 0.84  (0.07) | 334.67  (194.00) | 0.51  (0.02) | 0.80  (0.04) | 276.00  (124.85) |  | 0.48  (0.05) | 0.77  (0.08) | 653.33  (287.47) | 0.53  (0.01) | 0.76  (0.03) | 834.67  (170.63) |  | 0.46  (0.04) | 0.84  (0.12) | 409.00  (396.13) | 0.48  (0.09) | 0.93  (0.17) | 455.00  (249.68) |
|  | Post | 0.51  (0.02) | 0.81  (0.04) | 457.67  (171.83) | 0.49  (0.02) | 0.80  (0.03) | 512.67  (129.21) |  | 0.47  (0.07) | 0.85  (0.05) | 492.33  (396.80) | 0.54  (0.06) | 0.78  (0.04) | 801.00  (212.03) |  | 0.44  (0.02) | 0.95  (0.22) | 392.33  (421.85) | 0.47  (0.07) | 0.95  (0.05) | 679.00  (461.89) |

Supplementary Table 5. Results of the diffusion tensor tractography parameters for four neural tracts in three groups

This table presents the mean and standard deviation values of diffusion tensor tractography parameters for four neural tracts (CST, SST, Cingulum, and AF) in the UCB+EPO, UCB, and Control groups.

All data consist of a mean and standard deviation.

FA, fractional anisotropy, ADC, apparent diffusion coefficient, FN, fiber number, CST, corticospinal tract, SST, somatosensory tract, AF, arcuate fasciculus
