# Supplementary material for: Safety and efficacy of allogeneic umbilical cord blood cells and erythropoietin combination therapy in patients with subacute stroke
Source: Stem Cell Res Ther. 2025 Dec 27;17:56. doi: 10.1186/s13287-025-04856-8 (PMC12853616; doi:10.1186/s13287-025-04856-8)
Supplement: Supplementary file 12 — Supplementary material 12. [file 13287_2025_4856_MOESM12_ESM.docx]

Supplementary Table 6. Validation of cytokine level changes over time in the UCB+EPO, UCB, and control groups

|  | TNF-α | | | IL-1β | | | TGF-β | | | IL-8 | | |
| --- | --- | --- | --- | --- | --- | --- | --- | --- | --- | --- | --- | --- |
|  | UCB +EPO | UCB | Control | UCB +EPO | UCB | Control | UCB +EPO | UCB | Control | UCB +EPO | UCB | Control |
| D-day | 4.31 (6.38) | 0.85 (1.36) | 1.85 (2.14) | 15.42 (31.29) | 2.79 (5.75) | 0.49 (0.72) | 2.37 (1.79) | 1.37 (1.66) | 0.56 (0.27) | 13.35 (26.27) | 0.85 (0.94) | 1.69 (2.73) |
| D+1 | 1.92 (3.26) | 1.04 (1.17) | 2.99 (2.96) | 5.07 (5.85) | 1.37 (1.85) | 5.86 (8.42) | 2.16 (1.49) | 4.38 (7.07) | 1.04 (0.74) | 9.05 (11.24) | 1.07 (1.21) | 2.48 (4.01) |
| D+14 | 1.09 (0.96) | 2.48 (3.12) | 1.13 (0.82) | 4.97 (9.99) | 2.36 (3.16) | 0.23 (0.22) | 11.13 (15.67) | 1.49 (1.56) | 1.06 (0. 50) | 7.62 (7.09) | 1.12 (1.63) | 0.58 (0.45) |
| D+30 | 1.43 (2.44) | 0.36 (0.45) | 1.19 (0.60) | 3.38 (5.45) | 0.57 (1.01) | 0.25 (0.31) | 2.75 (3.11) | 1.10 (1.15) | 1.35 (1.63) | 6.58 (10.80) | 0.46 (1.01) | 0.86 (1.48) |

Quantitative real-time PCR for inflammation-related cytokines TNF-α, TGF-β, IL-1β, and IL-8 was analyzed in duplicate for each group containing five patients (each n=5).
Values represent fold changes relative to the day before therapy. Data are presented as mean (standard deviation).

D-day: The day of therapy, D+1: One day after therapy, D+14: 14 days after therapy, D+30: 30 days after therapy

UCB, Umbilical Cord Blood; EPO, Erythropoietin
